# Mechanistic investigation of glycolysis and pyroptosis in colon adenocarcinoma tissues, and prognostic analysis of patient clinical outcomes

**DOI:** 10.1371/journal.pone.0328560

**Published:** 2025-07-18

**Authors:** Yongling Wang, Zan Yuan, Yi Lao, Jiangtao He, Shufen Mo, Kangbiao Chen, Yanyan Ye, Lu Huang

**Affiliations:** 1 The First Department of Medical Oncology, Central Hospital of Guangdong Provincial Nongken, Zhanjiang Cancer Hospital, Zhanjiang, China; 2 Guangdong Medical University, Zhanjiang, China; 3 The Fourth Department of Medical Oncology, Central Hospital of Guangdong Provincial Nongken, Zhanjiang Cancer Hospital, Zhanjiang, China; 4 The Second Department of Medical Oncology, Central Hospital of Guangdong Provincial Nongken, Zhanjiang Cancer Hospital, Zhanjiang, China; Tianjin Medical University Cancer Institute and Hospital: Tianjin Medical University Cancer Institute & Hospital, CHINA

## Abstract

**Background:**

The exact mechanisms driving colorectal cancer (CRC) are yet to be fully elucidated. This study aims to confirm the reliability of a prognostic model for colon adenocarcinoma (COAD) by analyzing the varied expression levels of Glycolysis & Pyroptosis-Related Differentially Expressed Genes (G&PRDEGs) in COAD using bioinformatics tools.

**Methods:**

We retrieved gene expression data and clinical details for COAD patients from the Cancer Genome Atlas (TCGA) database. These data were analyzed to categorize the samples into pyroptosis-positive and pyroptosis-negative groups based on their expression of G&PRDEGs. A prognostic model for COAD was then developed using LASSO Cox regression analysis, focusing on these differentially expressed genes (DEGs). Kaplan-Meier curves were plotted to assess the differences in survival between the two groups. Furthermore, we conducted multivariate Cox regression analyses to evaluate the influence of clinical parameters and model-derived risk scores. Analyses of pathway enrichment were performed using R software, alongside single-sample gene-set enrichment analysis (ssGSEA) to explore the role of immune cells and functions associated with G&PRDEGs.

**Results:**

A predictive model was developed using 53 G&PRDEGs that were expressed differentially. An examination of survival rates revealed that the high-risk groups exhibited a noticeably diminished overall survival (OS) in comparison to the low-risk groups in the TCGA database (*P* < 0.001). An examination of the receiver operating characteristic (ROC) curve indicated that the risk score effectively predicted outcomes at 1, 3, and 5 years, with a space beneath the curve greater than 0.7. The risk score significantly affected the survival of COAD sufferers in the TCGA database, on the basis of the multivariate Cox regression analysis. The high-risk groups had a hazard ratio (HR) of 3.988 (95% CI 2.865 ~ 5.551, *P* < 0.001) in contrast to the low-risk groups. Examinations of enrichment identified an increase in the expression of DEGs in the high-risk groups, in contrast to the low-risk cohort, on the basis of the TCGA database. SsGSEA revealed elevated levels of immune cell soakage in the high-risk groups in contrast to the low-risk groups.

**Conclusion:**

The COAD prognosis model, developed using G&PRDEGs, exhibits predictive capability for the prognosis of COAD sufferers and offers utility in prognostic analysis for COAD sufferers.

## 1. Introduction

According to the World Health Organization’s International Agency for Research on Cancer (IARC), CRC [1] was the third most frequently diagnosed malignant tumor globally and the second leading cause of cancer-related mortality in 2022. CRC carcinogenesis is influenced by numerous risk factors, including genetic alterations, lifestyle choices, and environmental exposures. COAD, comprising over half of CRC cases, clinically manifests with alterations in bowel habits, bloody stools, abdominal distension, and localized pain, contributing to its high mortality rate. Despite ongoing research, the precise pathogenesis of COAD remains incompletely elucidated. Currently, clinical management mainly involves surgical resection supplemented by comprehensive treatments such as argeted therapy, chemotherapy, and immunosuppressive therapy, but the therapeutic effects are not entirely satisfactory. Investigating the mechanisms that underlie the emergence and growth of COAD and identifying key molecular markers are of paramount importance for early diagnostics and targeted molecular therapy of COAD.

This research appraises G&PRDEGs in COAD and assesses their association with somatic mutations (SM), copy number variations (CNV), and clinical outcomes. In this study, we performed an extensive bioinformatics analysis using COAD datasets from TCGA and the Gene Expression Omnibus (GEO). This included analyses such as Gene Ontology (GO), Kyoto Encyclopedia of Genes and Genomes (KEGG) pathway enrichment, gene set enrichment analysis (GSEA), and the creation of a prognostic risk model. Furthermore, immune infiltration and immunogenicity scores were assessed to investigate the potential of G&PRDEGs as targets for immunotherapy.

This study provides novel understandings of the molecular mechanisms behind COAD and emphasizes the prognostic and therapeutic significance of G&PRDEGs. Through the integration of multi-dimensional genomic data and advanced bioinformatic tools, we present a robust framework for identifying key molecular drivers and potential biomarkers in COAD, aiming to enhance patient outcomes through precision medicine.

## 2. Materials and methods

### 2.1. Data download

The TCGA database, which is accessible at https://portal.gdc.cancer.gov/, provided the COAD dataset, which we obtained employing the TCGAbiolinks [2] R package. The Colon Adenocarcinoma dataset (TCGA-COAD) was then evaluated as a test set. After removing data samples that lacked clinical data, we acquired all 462 COAD dates with clinical information and 39 Control samples in Counts format sequencing data. At the same time, it was standardized to the Fragments Per Kilobaseper Million (FPKM) structure, and the related clinical information was acquired through the UCSC Xena database [3] (https://xena.ucsc.edu/). The specific information is shown in Table 1.

**Table 1:** Overall baseline data sheet.

Characteristics	Overall
Age, n (%)	
> 60	322 (69.7%)
<= 60	140 (30.3%)
Gender, n (%)	
MALE	246 (53.2%)
female.	216 (46.8%)
Pathologic_stage, n (%)	
Stage IV	63 (14.0%)
Stage II	178 (39.5%)
Stage I	79 (17.5%)
Stage III	131 (29.0%)

Use the R program GEOquery [4] to obtain the COAD datasets [5], GSE20916 [6], and GSE44861 [7] originating from the GEO database (https://www.ncbi.nlm.nih.gov/geo/). These datasets will serve as a validation set for future validation purposes. The GSE20916 and GSE44861 samples were obtained from Homo sapiens and originated from colon tissue. The chip platforms of GSE20916 and GSE44861 were GPL570 and GPL3921, respectively, and the details are shown in Table 2. Among them, 30 colon tumors in dataset GSE20916 were selected as COAD samples and 34 normal tumors as Control samples. Dataset GSE44861 contains 56 tumors as COAD samples and 55 adjacent nontumor samples as Control samples. All selected COAD specimens and control specimens were incorporated into this research.

**Table 2:** GEO microarray chip information.

	GSE20916	GSE44861
Platform	GPL570	GPL3921
Species	Homo sapiens	Homo sapiens
Tissue	Colon Tissues	Colon Tissues
Samples in COAD group	30	56
Samples in Control group	34	55
Reference	PMID: 20957034	PMID: 23982929

GEO, Gene Expression Omnibus; COAD, Colon Adenocarcinoma.

The GeneCards database [8] (https://www.genecards.org/) is a collection of glycolysis and pyroptosis-related genes (Glycolysis & Pyroptosis – Related Genes, G&PRGs). An extensive amount of information pertaining to human genes can be found in the GeneCards database. We employed the phrases “Glycolysis” and “Pyroptosis” as keywords for searches, respectively, and retained just “protein-coding” glycolysis-related genes (GRGs). The sum of, 3293 GRGs was obtained. After the exclusion of non-protein-coding genes, we identified 534 pyroptosis-related genes (PRGs). Furthermore, a comprehensive set of 291 genes associated with glycolysis referred to as GRGs was acquired from the published literature [9] by utilizing the keyword “glycolysis” in PubMed. The sum of 52 PRGs was [10] acquired from PubMed using “pyroptosis” as the search term. The sum of 3393 GRGs was acquired by deduplication and merging of the glycolysis-related genes obtained in the above way. After performing a combined deduplication of pyroptosis-related genes, a total of 541 PRGs were found. Finally, GRGs and PRGs were intersected to obtain 308 G&PRGs. The complete dataset is detailed in S1 Table.

The datasets GSE20916 and GSE44861 were debatched using the R package sva [11]. The datasets GSE20916 and GSE44861 were normalized with the R package limma [12], ensuring uniformity across the annotation probes. The study comprised all the chosen samples from dataset GSE20916 as the validation set, while dataset GSE44861 was utilized as the validation set for further analysis.

### 2.2. Genes exhibiting differential expression in the context of both glycolysis and pyroptosis in relation to COAD

To identify DEGs between COAD samples and control groups, we employed the R package limma. We set thresholds for DEGs at |logFC| > 0.5 and adj. P < 0.05, classifying those with |logFC| > 0.5 as upregulated and those with |logFC| < −0.5 as downregulated. The Benjamini-Hochberg (BH) method was utilized to adjust p-values for multiple testing. Visualizations, including volcano plots, were generated using the R package ggplot2 to represent the DEG results.

According to the sample grouping of data sets GSE20916 and GSE44861, the samples were split up into COAD specimens and control specimens, respectively. In further analysis, the limma package was applied to assess DEGs in COAD and control samples with adjusted thresholds of |logFC| > 0 and P < 0.05. Genes with |logFC| > 0 and P < 0.05 were identified as upregulated, while those with |logFC| < 0 and P < 0.05 were designated as downregulated.

To acquire G&PRDEGs connected to COAD, the colon adenocarcinoma data set (TCGA-COAD) is obtained by variance analysis of all | logFC | > 0.5 and adj. *P* < 0.05 DEGs; the GSE20916 data sets and GSE44861 are obtained by variance analysis of all | logFC | > 0 and *P* < 0.05 DEGs; and G&PRGs get intersection and map Venn, G&PRDEGs related to cells, using the R package pheatmap to create a heat map visualization.

### 2.3. Analysis of CNV and SM

To examine SM in the TCGA-COAD, the SM data of the COAD group in the Cancer Genome Project (TCGA) were chosen as the masked somatic mutation data, and the data underwent processing using the VarScan software. The mutation spectrum of SM was delineated using the R package tools [13].

To analyze CNV in the COAD group of the TCGA-COAD, the “Masked Copy Number Segment” data was selected as the CNV data of the COAD group of the TCGA-COAD. Then, we performed GISTIC2.0 [14] analysis on the obtained and handled CNV segments, and all the default parameters were used in the GISTIC2.0 analysis.

### 2.4. Analysis of GO and KEGG pathways enrichment

Functional enrichment analyses were conducted using the GO method [15] to explore biological processes (BP), cellular components (CC), and molecular functions (MF). The KEGG database [16] was employed for comprehensive pathway and drug-target studies. These analyses were facilitated by the R package clusterProfiler [17]. The screening criteria were set with a statistical significance threshold of P < 0.05 and an FDR value (q value) < 0.25. Visualization of the pathway maps from the KEGG pathway enrichment analysis was achieved using the R package Pathview [18].

### 2.5. GSEA

The GSEA [19] algorithm was applied to assess gene distribution patterns within specific gene sets, ordered by their correlation to the phenotype, to understand their impact on the phenotype. Initially, genes from the TCGA-COAD dataset were ranked based on their logFC values, and then the R package clusterProfiler was utilized to perform GSEA on all TCGA-COAD genes. Parameters for the GSEA included a seed value of 2020 and a calculation count of 1000. Each gene set in the analysis was required to contain at least 10 genes but no more than 500 genes. Gene sets c2 were accessed via the Molecular Signatures Database (MSigDB) [20], and each symbol from the 2022.1 edition of the Handbook was compared. The GMT file, which includes all 3050 Canonical Pathways, was utilized in the GSEA. The criteria for GSEA included an adjusted P-value < 0.05 and an FDR below 0.25, with p-value correction carried out using the BH method.

### 2.6. Building a COAD prognostic risk model

To develop the prognostic risk model for the TCGA-COAD dataset, the survival R package was employed. Clinical data were analyzed to evaluate the prognostic significance of G&PRDEGs and their potential as independent predictors. Initially, univariate Cox regression analysis was performed, with variables showing a P-value below 0.10 selected for subsequent LASSO regression analysis. The outcomes from the univariate Cox regression were represented through a forest plot.

Whereafter, LASSO regression analysis was completed utilizing the R package glmnet [21] with family = “cox” as the parameter based on the G&PRDEGs merge into the univariate Cox regression analysis, and the quantity of cycles was set to 10. LASSO regression analysis is predicated on linear regression analysis; by adding a penalty term (lambda × absolute value of slope), the overfitting of the model is lowered, and the model’s capacity for generalization is improved. The results from the LASSO regression were illustrated with diagrams of the prognostic risk model and variable trajectories. Finally, multivariate Cox regression analysis was conducted, using the LASSO Risk Score and clinical data to identify crucial genes within the predictive risk model. The risk variables associated with the LASSO risk score were visually depicted using the ggplot2 R program. The risk score was computed with the accompanying formula:


$$\text{risk}Score\;=\;\sum_{i}Coefficient\;\left({gene}_{i}\right)*mRNA\;Expression\;({gene}_{i})$$

A study was also conducted to compare overall survival (OS) between low-risk and high-risk COAD groups within the TCGA-COAD dataset. Kaplan-Meier (KM) curve analysis [22] was performed using the survival R package, with the KM curve generated based on the LASSO Risk Score. The time-dependent ROC curve [23] is a visual tool that helps to identify optimal models, discard less effective ones, or determine the best threshold within a model. The survival ROC R package was used to create a time-dependent ROC curve and calculate the area under the curve (AUC) based on the risk score and overall survival derived from LASSO. Predictions for 1, 3, and 5-year survival outcomes were made for the COAD cohort within TCGA-COAD. The AUC values range from 0.5 to 1, where higher values closer to 1 indicate better diagnostic performance. An AUC greater than 0.5 indicates a likelihood of molecular expression promoting the event, with values between 0.5 to 0.7 indicating low accuracy, 0.7 to 0.9 moderate accuracy, and above 0.9 high accuracy.

### 2.7. Prognostic analysis of COAD prognostic risk model

The results of the multivariate Cox regression analysis were presented using a forest plot, showing the expression levels of the risk score and associated clinical data. A nomogram [24], constructed with the “rms” R package, visually maps the relationships among multiple independent variables, focusing on the relationship between the risk score and clinical data.

The calibration curve plots the true versus predicted probabilities under different conditions to assess the model’s predictive accuracy. This curve was developed through a detailed calibration analysis, evaluating both the precision and discrimination abilities of the prognostic risk model based on the LASSO-derived risk score. Decision Curve Analysis (DCA) [25], supported by the ggDCA R package, was utilized to create a DCA diagram using the LASSO Risk Score. This analysis measures the clinical utility of molecular markers across various clinical settings, predictive models, and diagnostic evaluations.

### 2.8. Verification of differential expression of key genes and ROC curve analysis

We examined the gene expression variations between the COAD group and the control group using the TCGA-COAD dataset, along with GSE20916 and GSE44861. To facilitate this, group comparison maps were created highlighting the differences driven by selected gene expression levels. ROC curves were generated for key genes using the pROC R package, and the Area Under the Curve (AUC) was calculated to assess the impact of these gene expression levels on COAD progression diagnostics. The AUC values range from 0.5 to 1, with higher values indicating better diagnostic performance. Specifically, AUC values between 0.5 to 0.7 suggest low accuracy, 0.7 to 0.9 indicate moderate accuracy, and values above 0.9 indicate high diagnostic accuracy.

Subsequently, the Spearman correlation analysis was employed to explore the relationships among key genes across the TCGA-COAD, GSE20916, and GSE44861 datasets. We visualized these relationships through a correlation heatmap, produced using the R package “heatmap”. Correlation coefficients below 0.3 indicate a weak or negligible relationship, those ranging from 0.3 to 0.5 suggest a mild correlation, values between 0.5 and 0.8 signify a moderate association, and coefficients above 0.8 denote a strong correlation.

The process of comparing GO annotations allows for a quantitative approach to determining the likeness of genes and genomes. This method has gained significance as a foundation for many bioinformatics analytic techniques. The GOSemSim [26] R program was utilized to compute the functional correlation of key genes. Subsequently, they were compared using functional similarity (friends). Ultimately, the R package RCircos [27] was hired to visually show the precise position of key genes on the chromosome.

### 2.9. GSVA

The GSVA [28] serves as a nonparametric and unsupervised technique for evaluating gene set enrichment from microarray nuclear transcriptomes, converting gene expression data across various samples into a comparative matrix. This method aims to explore pathway enhancements across these samples. Using the h.all.v7.4.symbols.gmt gene set from the Molecular Signatures Database (MSigDB) [20], a gene set variation analysis (GSVA) was performed on the entire gene pool in the TCGA-COAD dataset. This analysis focused on identifying differences in functional enrichment between low- and high-risk groups, with a significance threshold of P < 0.05.

### 2.10. Evaluation of the immunogenicity score (IPS)

The ssGSEA [29] method quantifies the comparatively high abundance of immunocyte infiltrates. In the initial phase, various immune cell types were classified, including activated CD8 + T cells, activated dendritic cells, gamma-delta T cells, natural killer cells, and several human immune cell subsets such as regulatory T cells. Subsequently, ssGSEA analysis provided enrichment scores to estimate the relative levels of immune cell infiltration in each sample. An infiltration matrix for COAD samples from the TCGA-COAD dataset was compiled, and the R package ggplot2 was used to create visual group comparisons, highlighting immune cell expression differences between low- and high-risk COAD groups. Selected immune cells with significant differences were further analyzed. The relationships between immune cells and key genes were explored using the Spearman correlation method, with the results depicted in a bubble plot generated using ggplot2.

### 2.11. IPS analysis

Immunogenicity refers to the ability of an antigen or its epitopes to act on antigen recognition receptors of T cells and B cells to elicit humoral and/or cell-mediated immune responses. This principle of immunity could be called immunogen. Immunogenicity is determined by several genes, including those associated with effector cells, MHC molecules, immunomodulatory factors, and immunosuppressive cells. Machine learning techniques enable the estimation and quantification of immunogenicity. The Cancer Immunome Atlas (TCIA) database [30] offers immunoscores (IPS) for 20 different cancers, which serve as reliable indicators of PD-1 and CTLA-4 responses. Additionally, IPS data for COAD samples were retrieved from TCGA-COAD, and comparisons of IPS between low- and high-risk groups were visualized and analyzed for differences using ggplot2.

### 2.12. Statistical analysis

Data processing and statistical analyses were carried out using R software (Version 4.2.2). For variables with normal distribution not specifically mentioned, the independent Student’s T-Test was used to evaluate significance when comparing continuous variables between two groups. For non-normally distributed variables, the Mann-Whitney U Test, also known as the Wilcoxon Rank Sum Test, was applied. For analyses involving three or more groups, the Kruskal-Wallis test was used. Spearman’s correlation analysis was performed to determine correlation coefficients among various compounds. Statistical analyses were based on two-sided p-values, with significance set at p < 0.05 unless otherwise specified.

## 3. Results

### 3.1. Technology roadmap

**Fig 1:**
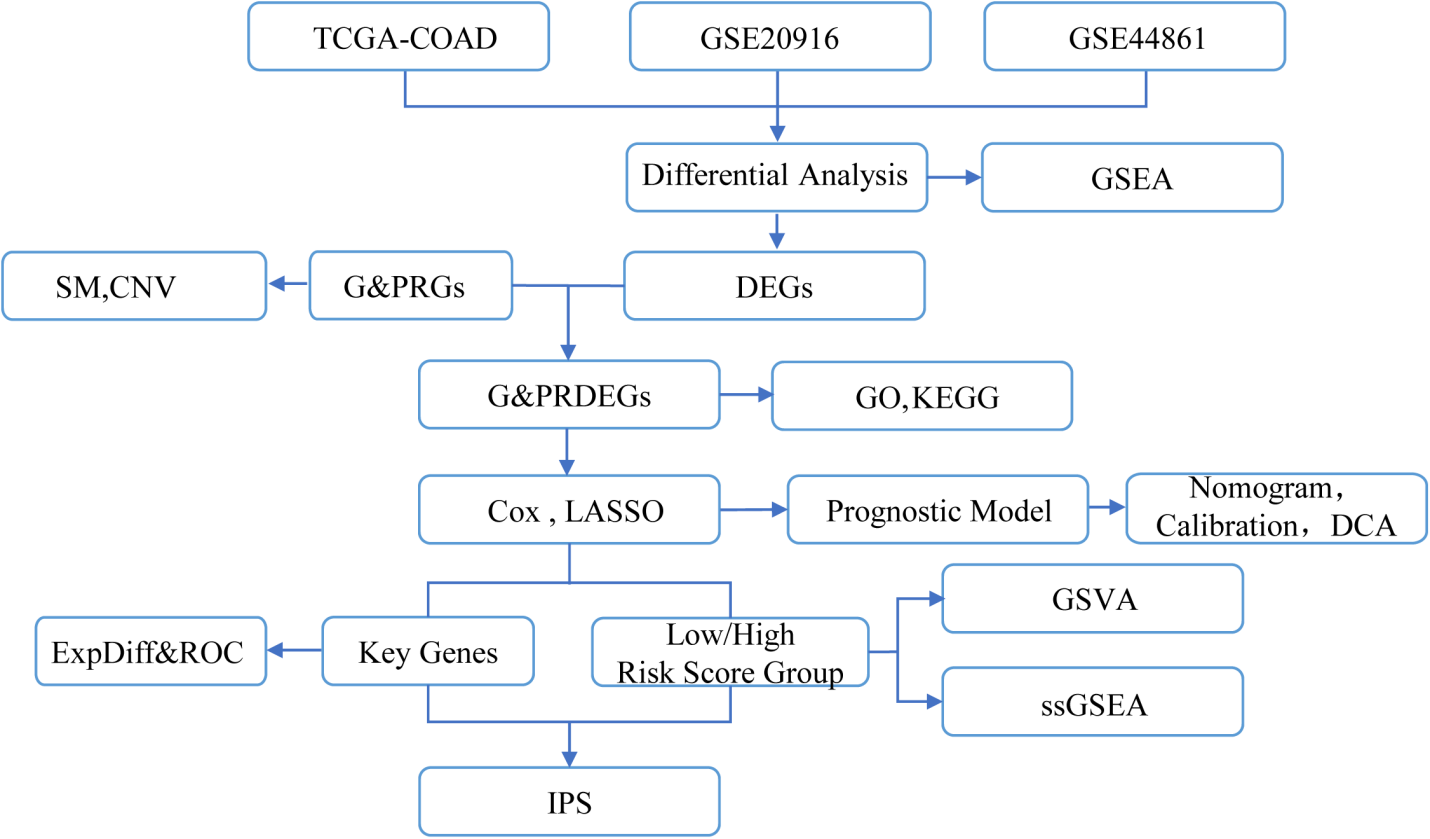
Flow chart for the comprehensive analysis. TCGA, The Cancer Genome Atlas; COAD, Colon Adenocarcinoma; DEGs, Differentially Expressed Genes; G&PRGs, Glycolysis & Pyroptosis-Related Genes; G&PRDEGs, Glycolysis & Pyroptosis-Related Differentially Expressed Genes; GSEA, Gene Set Enrichment Analysis; GO, Gene Ontology; KEGG, Kyoto Encyclopedia of Genes and Genomes; SM, Somatic Mutation; CNV, Copy Number Variations; ROC, Receiver Operating Characteristic; LASSO, Least Absolute Shrinkage and Selection Operator; DCA, Decision Curve Analysis; GSVA, Gene Set Variation Analysis; ssGSEA, single-sample Gene-Set Enrichment Analysis; IPS, Immunophenoscore.

This study involved the collection and analysis of datasets from TCGA-COAD, GSE20916, and GSE44861. We utilized differential analysis for data processing, followed by correlation analysis. The study’s workflow is depicted in Fig 1.

### 3.2. Processing of COAD datasets

**Fig 2:**
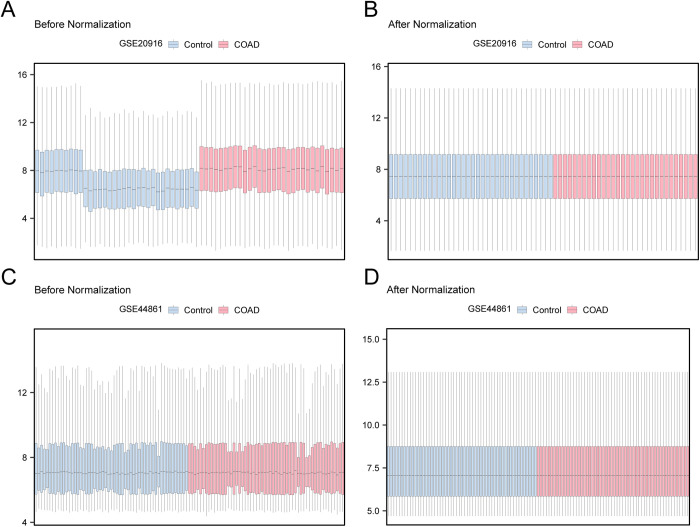
Batch Effects Removal of GSE20916 And GSE44861. A. Box plot of the GSE20916 distribution of the dataset before batch removal. B. The boxplot of the GSE20916 distribution of the dataset after going to batch. C. Distribution boxplot of dataset GSE44861 before debatching. D. Distribution boxplot of the dataset GSE44861 after debatching. COAD, Colon Adenocarcinoma. Light blue is the Control group and light red is the COAD group.

Initially, batch effects were removed from the GSE20916 and GSE44861 COAD datasets using the R package sva. Afterward, the distribution boxplot (Fig 2A–D) was used to compare the values of expression of the datasets both prior to and following the batch effect removal. Following batch removal, the distribution boxplot results demonstrated that the batch effect of the specimens in the COAD dataset was essentially removed.

### 3.3. COAD related glycolysis and pyroptosis corresponding genes with differential expression

**Fig 3:**
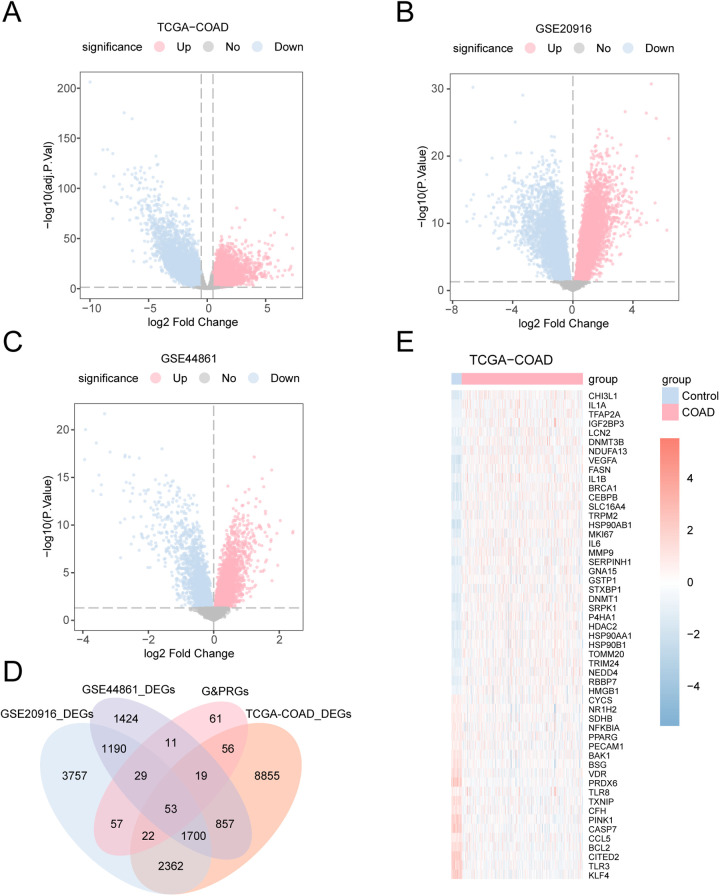
Differential gene expression analysis. A. Volcano plot of DEGs analysis between COAD specimens and control specimens in the colon adenocarcinoma dataset (TCGA-COAD). B. Volcano plot of DEGs analysis of COAD specimens and control specimens in dataset GSE20916. C. Volcano plot of DEGs analysis of COAD specimens and control specimens in dataset GSE44861. D. Differentially expressed genes (DEGs) in the colon adenocarcinoma dataset (TCGA-COAD), differentially expressed genes (DEGs), and glycolysis and pyroptosis-related genes (G&PRGs) Venn diagram in datasets GSE20916 and GSE44861. E. Heat map of differentially expressed genes (G&PRDEGs) related to glycolysis and pyroptosis in the colon adenocarcinoma dataset (TCGA-COAD). TCGA, The Cancer Genome Atlas; COAD, Colon Adenocarcinoma; DEGs, Differentially Expressed Genes; G&PRGs, Glycolysis & Pyroptosis-Related Genes; G&PRDEGs, Glycolysis & Pyroptosis-Related Differentially Expressed Genes. In the heat map grouping, the light red is the COAD sample and the light blue is the Control sample. Red represents high expression and blue represents low expression in the heat map.

TCGA-COAD data were categorized into COAD and control specimens. Differential analysis using the R package limma was conducted on TCGA-COAD and datasets GSE20916 and GSE44861, aiming to pinpoint gene expression variations. Results indicated that for TCGA-COAD, 13924 genes exceeded the DEGs threshold of |logFC| > 0.5 with an adjusted P < 0.05 (Fig 3A). Specifically, 7786 genes showed increased expression and 6138 showed decreased expression. For dataset GSE20916, 9170 genes crossed the DEGs threshold of |logFC| > 0 and P < 0.05, with 4956 showing higher expression and 4214 showing lower expression (Fig 3B). Dataset GSE44861 recorded 5283 DEGs, with 2856 upregulated and 2427 downregulated genes, according to the dataset map volcano figures(Fig 3C).

To isolate G&PRDEGs, datasets were filtered to include only those genes with significant differential expression (|logFC| > 0.5, adj. P < 0.05 for TCGA-COAD and |logFC| > 0, P < 0.05 for GSE20916 and GSE44861). The intersection of DEGs across these datasets was visualized using a Venn diagram (Fig 3D), revealing 53 unique G&PRDEGs (see Table 2 for details), specific information see S2 Table. Further, DEGs associated with G&PRDEGs were analyzed across different TCGA-COAD sample groups, and their intersections were visualized using a heatmap generated with the pheatmap R package (Fig 3E).

### 3.4. Glycolysis & pyroptosis related gene CNV, SM

**Fig 4:**
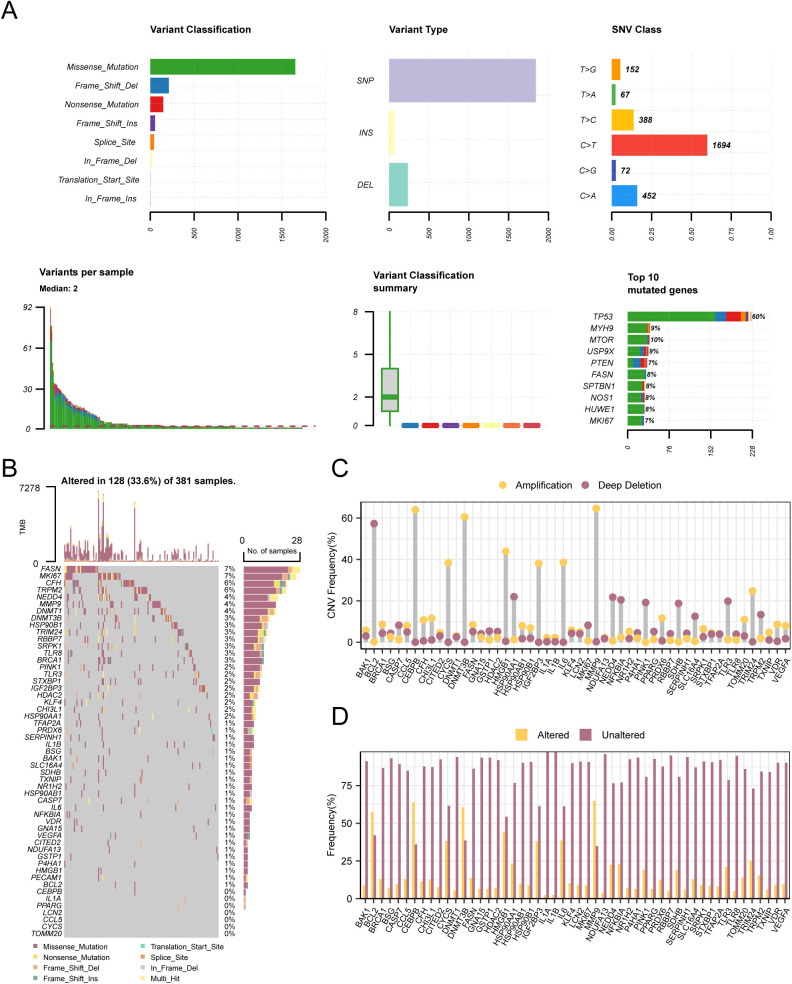
CNV and SM analysis. A. Presentation of somatic mutations (SM) of glycolysis & pyroptosis-related genes (G&PRGs) in COAD samples from the Colon Adenocarcinoma Dataset (TCGA-COAD). B. Presentation of somatic mutations (SM) of glycolysis & pyroptosis-associated differential genes (G&PRDEGs) in COAD samples from the Colon Adenocarcinoma Dataset (TCGA-COAD). C-D. The copy number variation (CNV) profile of glycolysis & pyroptosis-associated differential genes (G&PRDEGs) in COAD samples from the Colon Adenocarcinoma Dataset (TCGA-COAD) is shown. TCGA, The Cancer Genome Atlas; COAD, Colon Adenocarcinoma; G&PRGs, Glycolysis & Pyroptosis-Related Genes; G&PRDEGs, Glycolysis & Pyroptosis-Related Differentially Expressed Genes; SM, Somatic Mutation; SNV, Single Nucleotide Variant; SNP, Single Nucleotide Polymorphism; CNV, Copy Number Variations.

To analyze the SM of 308 G&PRGs in COAD samples from the TCGA-COAD dataset, mutation analysis results of these genes were tabulated and visualized using R package tools (Fig 4A). The findings took out that there were eight primary categories of SM in genes associated with G&PRGs, with missense mutations comprising the largest proportion. In addition, the mutation type of 308 G&PRGs in COAD samples was mainly single nucleotide polymorphism (SNP). In addition, the C-to-T mutation was the single nucleotide variant (SNV) that was most prevalent in COAD samples. Next, we also analyzed the SM status of 53 G&PRDEGs in COAD samples and ranked them according to mutation frequency, from high to low. Fifty-three G&PRDEGs were visualized (Fig 4B). The results showed that G&PRDEGs *FASN* had the highest mutation rate, with a mutation rate of 7%.

To analyze the CNVS of G&PRDEGs in COAD samples from the TCGA-COAD dataset, we extracted and integrated the COAD sample CNV data from the TCGA-COAD and analyzed them by GISTIC2.0. A total of 53 G&PRDEGs were found to have CNVS in COAD samples, and then the mutation status of 53 genes with CNV was shown (Fig 4C, D). The genes included: *BAK1, BCL2, BRCA1, BSG, CASP7, CCL5, CEBPB, CFH, CHI3L1, CITED2, CYCS, DNMT1, DNMT3B, FASN, GNA15, GSTP1, HDAC2, HMGB1, HSP90AA1, HSP90AB1, HSP90B1, IGF2 BP3, IL1A, IL1B, IL6, KLF4, LCN2, MKI67, MMP9, NDUFA13, NEDD4, NFKBIA, NR1H2, P4HA1, PECAM1, PINK1, PPARG, PRDX6, RBBP7, SDHB, SERPINH1, SLC16A4, SRPK1, STXBP1, TFAP2A, TLR3, TLR8, TOMM20, TRIM24, TRPM2, TXNIP, VDR, VEGFA.*

### 3.5. Evaluation of enrichment in KEGG pathways

**Fig 5:**
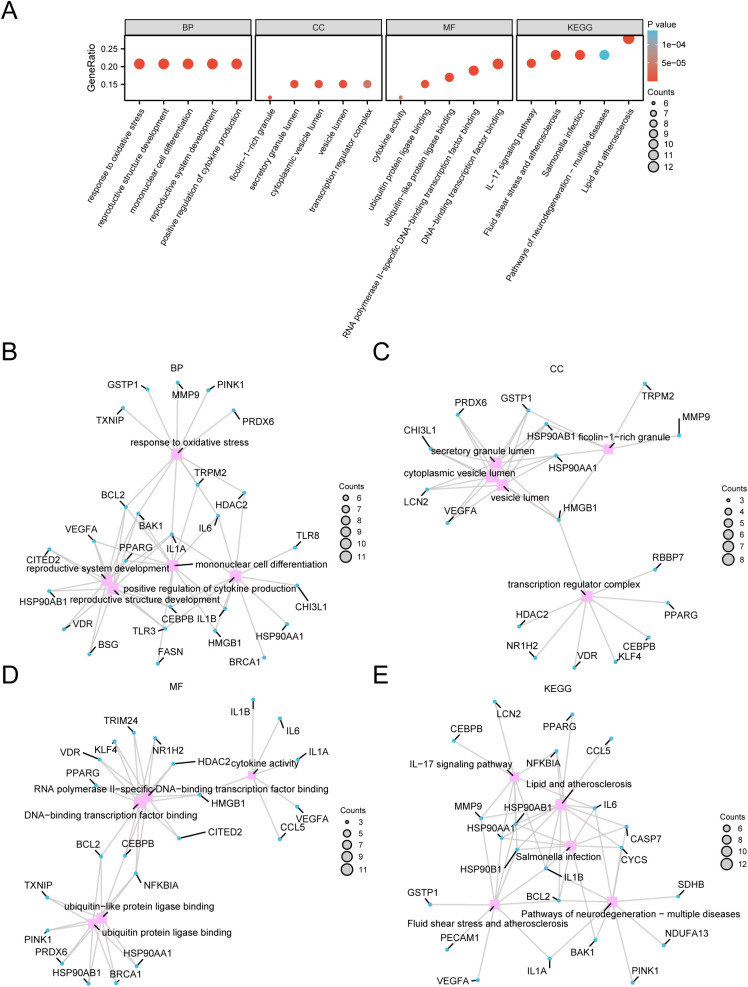
GO and KEGG enrichment analysis for G&PRDEGs. A. Bubble plot of gene ontology (GO) and Kyoto Encyclopedia of Genes and Genomes (KEGG) enrichment analysis results of glycolysis & pyroptosis-related differentially expressed genes (G&PRDEGs): biological process (BP), cell component (CC), molecular function (MF) and biological KEGG pathway. GO terms and KEGG terms are shown on the abscissa. B-e. Gene ontology (GO) and Kyoto Encyclopedia of Genes and Genomes (KEGG) enrichment analysis results of glycolysis & pyroptosis-related differentially expressed genes (G&PRDEGs) network diagram showing BP (B), CC (C), MF (D) and KEGG (E). Pink nodes represent items, blue nodes represent molecules, and lines represent the relationship between items and molecules. G&PRDEGs, Glycolysis & Pyroptosis-Related Differentially Expressed Genes; GO, Gene Ontology; KEGG, Kyoto Encyclopedia of Genes and Genomes; BP, Biological Process; CC, Cellular Component; MF, Molecular Function. The bubble size in the bubble plot represents the number of genes, and the color of the bubble represents the size of the p-value value. The redder the color, the smaller the p-value, and the bluer, the larger the p-value. The screening criteria for gene ontology (GO) and Kyoto Encyclopedia of Genes and Genomes (KEGG) enrichment analysis were *P* < 0.05 and FDR value (q value) < 0.25.

GO and KEGG pathway analyses were used to investigate the relationships between the BP, CC, MF, and biological KEGG pathways of the 53 DEGs associated with glycolysis and pyroptosis in relation to COAD. Table 3 displays the findings of the KEGG pathways and enrichment analysis using the 53 DEGs related to G&PRDEGs. The analyses revealed 53 genes associated with glycolysis and pyroptosis. These genes, known as G&PRDEGs, were primarily shown to be enriched in COAD. A study was carried out in COAD to explore how oxidative stress impacts the development of reproductive structures and to conduct enrichment analysis of KEGG pathways. BP focused on the development of reproductive systems, mononuclear cell differentiation, and cytokine production enhancement; CC included secretory granule lumen and cytoplasmic vesicle lumen; MF involved the binding of transcription factors to DNA, specific interactions of RNA polymerase II, and cytokine activity. The sample exhibited high levels of fluid shear stress and atherosclerosis, lipids and atherosclerosis, and Salmonella infection. Additionally, it showed enrichment in the pathways of neurodegeneration and multiple illnesses and the IL-17 signaling KEGG pathway. KEGG pathways augmentation analysis findings were displayed using bubble plots (Fig 5A).

**Table 3:** Results of GO and KEGG enrichment analysis for G&PRDEGs.

ONTOLOGY	ID	Description	GeneRatio	BgRatio	p value	p.adjust	q value
BP	GO:0006979	response to oxidative stress	11/53	433/18800	2.721 e-08	9.6509 e-06	4.9526 e-06
BP	GO:0048608	reproductive structure development	11/53	433/18800	2.721 e-08	9.6509 e-06	4.9526 e-06
BP	GO:1903131	mononuclear cell differentiation	11/53	433/18800	2.721 e-08	9.6509 e-06	4.9526 e-06
BP	GO:0061458	reproductive system development	11/53	436/18800	2.9201 e-08	9.6509 e-06	4.9526 e-06
BP	GO:0001819	positive regulation of cytokine production	11/53	475/18800	6.9819 e-08	1.6782 e-05	8.6122 e-06
CC	GO:0034774	secretory granule lumen	8/53	322/19594	2.2719 e-06	0.00014434	9.1159 e-05
CC	GO:0060205	cytoplasmic vesicle lumen	8/53	325/19594	2.4338 e-06	0.00014434	9.1159 e-05
CC	GO:0031983	vesicle lumen	8/53	327/19594	2.5471 e-06	0.00014434	9.1159 e-05
CC	GO:0005667	transcription regulator complex	8/53	483/19594	4.3044 e-05	0.00073174	0.00046215
CC	GO:0101002	ficolin-1-rich granule	6/53	185/19594	1.0374 e-05	0.00031932	0.00020167
MF	GO:0140297	DNA-binding transcription factor binding	11/53	470/18410	7.7404 e-08	1.1224 e-05	8.0255 e-06
MF	GO:0061629	RNA polymerase II-specific DNA-binding transcription factor binding	10/53	348/18410	4.8507 e-08	1.1224 e-05	8.0255 e-06
MF	GO:0044389	ubiquitin-like protein ligase binding	9/53	317/18410	2.706 e-07	2.6158 e-05	1.8705 e-05
MF	GO:0031625	ubiquitin protein ligase binding	8/53	298/18410	2.0208 e-06	0.00014651	0.00010476
MF	GO:0005125	cytokine activity	6/53	235/18410	5.6427 e-05	0.00163639	0.00117012
KEGG	hsa05417	Lipid and atherosclerosis	12/43	215/8164	6.124 e-10	8.0267 e-08	4.717 e-08
KEGG	hsa05418	Fluid shear stress and atherosclerosis	10/43	139/8164	1.7568 e-09	9.5452 e-08	5.6094 e-08
KEGG	hsa05132	Salmonella infection	10/43	249/8164	4.5949 e-07	1.498 e-05	8.803 e-06
KEGG	hsa05022	Pathways of neurodegeneration – multiple diseases	10/43	476/8164	0.00013836	0.00112764	0.00066268
KEGG	hsa04657	IL-17 signaling pathway	9/43	94/8164	9.8487 e-10	8.0267 e-08	4.717 e-08

GO, Gene Ontology; BP, Biological Process; CC, Cellular Component; MF, Molecular Function; KEGG, Kyoto Encyclopedia of Genes and Genomes; G&PRDEGs, Glycolysis & Pyroptosis-Related Differentially Expressed Genes.

Meanwhile, network diagrams depicting CC, BP, MF, and biological KEGG pathways were created based on the results from GO and KEGG pathway enrichment analyses (Fig 5B–E). The lines depict the molecules that correspond to the entries, together with their respective annotations. The extent of the nodes indicates the quantity of molecules included in each entry.

### 3.6. GSEA

**Fig 6:**
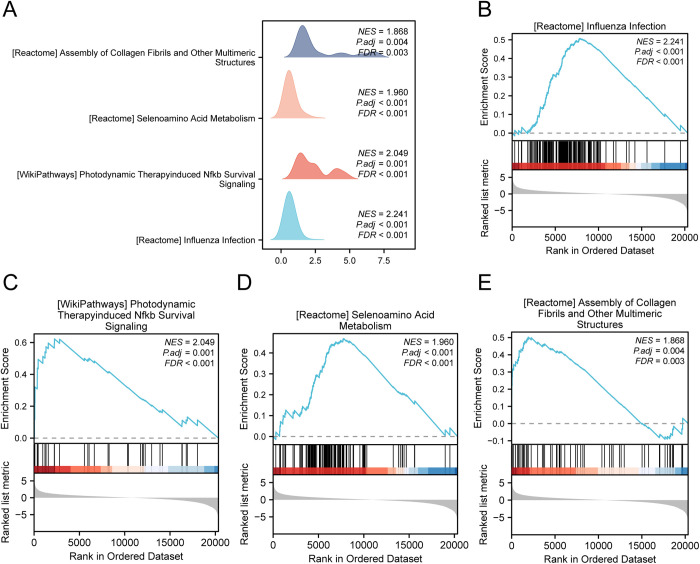
GSEA for TCGA-COAD. A. Presentation of 4 biological function mountain maps from GSEA of the colon adenocarcinoma dataset (TCGA-COAD). B-e. Gene set enrichment analysis (GSEA) showed that all genes were significantly enriched in influenza infection (B), photodynamic therapy-induced Nfkb survival signaling (C), influenza infection (B) and NFKB survival signaling (C). Selenoamino Acid Metabolism (D) and the Assembly of Collagen Fibrils and Other Multimeric Structures (E). GSEA, Gene Set Enrichment Analysis; TCGA, The Cancer Genome Atlas; COAD, Colon Adenocarcinoma. The screening criteria of gene set enrichment analysis (GSEA) were adj.p < 0.05 and FDR value (q value) < 0.25, and the p value correction method was BH.

To understand the impact of gene expression on COAD, GSEA was employed to analyze gene expression and functions within the TCGA-COAD dataset. Detailed results are presented in Table 4, which includes the relationship between molecular functions and affected cellular components (Fig 6A). The analysis revealed significant increases in gene activity associated with influenza infection (Fig 6B), Nfkb signaling induced by photodynamic therapy (Fig 6C), selenium acid metabolism (Fig 6D), and the assembly of collagen fibrils and other complex structures (Fig 6E), among other functions and pathways.

**Table 4:** Results of GSEA for TCGA-COAD.

ID	setSize	enrichmentScore	NES	p value	p.adjust	q value
REACTOME_INFLUENZA_INFECTION	156	0.50752594	2.24052592	1E-10	6.6215 e-09	4.924 e-09
WP_PHOTODYNAMIC_THERAPYINDUCED_NFKB_SURVIVAL_SIGNALING	34	0.62274719	2.04903893	6.4721 e-05	0.00104873	0.00077988
REACTOME_SELENOAMINO_ACID_METABOLISM	117	0.46855192	1.96010399	8.9576 e-07	2.4789 e-05	1.8434 e-05
REACTOME_ASSEMBLY_OF_COLLAGEN_FIBRILS_AND_OTHER_MULTIMERIC_STRUCTURES	61	0.50260173	1.86783658	0.00031935	0.00390793	0.00290608

TCGA, The Cancer Genome Atlas; COAD, Colon Adenocarcinoma; GSEA, Gene Set Enrichment Analysis.

### 3.7. Building a COAD predictive risk model

**Fig 7:**
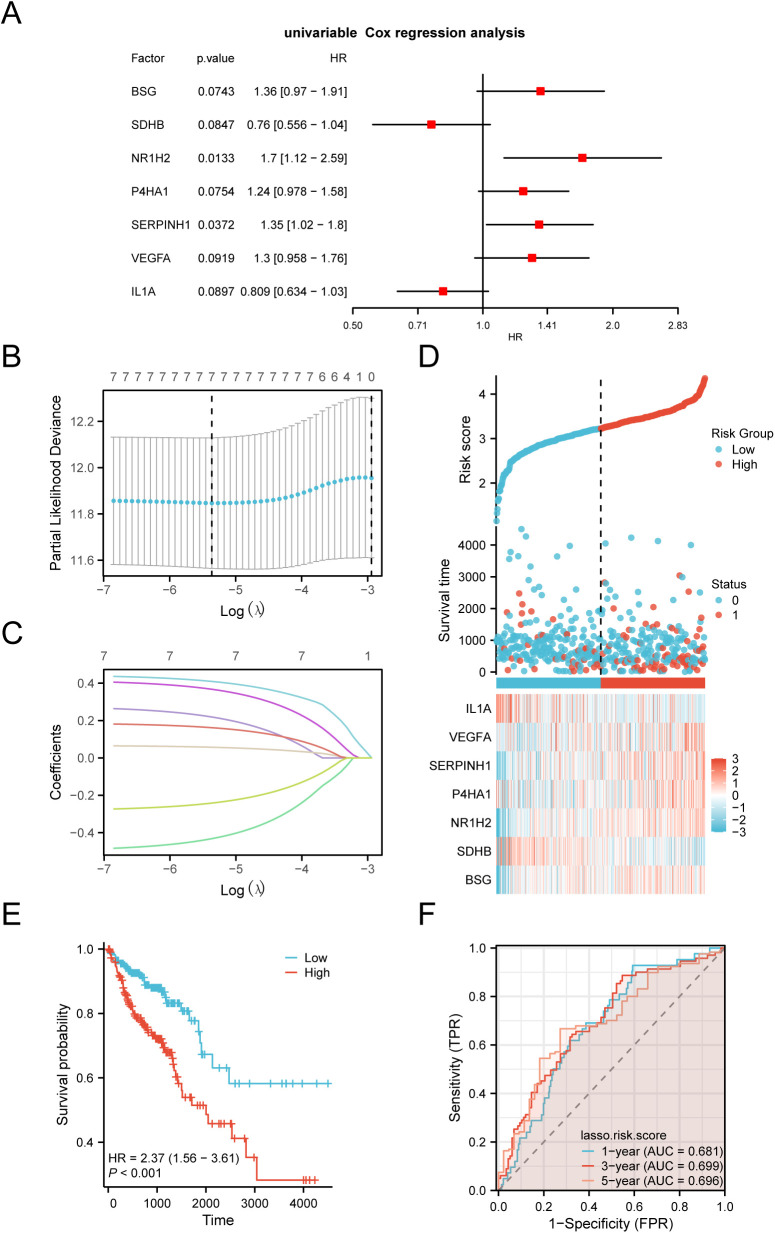
LASSO and cox regression analysis. A. Forest plot of glycolysis & pyroptosis-related differentially expressed genes (G&PRDEGs) by univariate Cox regression analysis of prognosis. B-c. Prognostic risk model plot (B) and variable trajectory plot (C) of the LASSO regression model. D. Risk factor map of the prognostic LASSO model for key genes. E. Prognostic KM curves between high and low Risk Score groups of LASSO and OS in the COAD group. F. Time-dependent ROC curve of the COAD group in the Colon Adenocarcinoma dataset (TCGA-COAD). G&PRDEGs, Glycolysis & Pyroptosis-Related Differentially Expressed Genes; LASSO, Least Absolute Shrinkage and Selection Operator; OS, Overall Survival; KM, Kaplan-Meier; ROC, Receiver Operating Characteristic Curve; AUC, Area Under the Curve; TPR, True Positive Rate; FPR, False Positive Rate; TCGA, The Cancer Genome Atlas; COAD, Colon Adenocarcinoma. *** represents *P* < 0.001, highly statistically significant. When AUC > 0.5, it indicates that the expression of the molecule is a trend to promote the occurrence of the event, and the closer the AUC is to 1, the better the diagnostic effect. AUC values between 0.5 and 0.7 were associated with lower accuracy.


Building the COAD prognostic risk model, we applied the clinical data of the COAD group in the colon cancer dataset (TCGA-COAD) combined with G&PRDEGs to carry out a Cox regression analysis in univariate. After initial univariate analysis, variables showing P < 0.10 were selected for inclusion in the LASSO regression analysis, which was illustrated using a forest plot (Fig 7A). For a better assessment of the predictive power of specific genes in COAD, a LASSO regression model was developed, and visualizations of the model map (Fig 7B) and variable trajectories (Fig 7C) were produced. The outcomes demonstrated that the LASSO regression model contained 7 LASSO regression model genes, namely: *BSG, SDHB, NR1H2, P4HA1, SERPINH1, VEGFA and IL1A*. Define them as key genes:

RiskScore =BSG*0.2188 −SDHB*0.4316 +NR1H2*0.4092+P4HA1*0.3672 +SERPINH1*0.0572 +VEGFA*0.1618 −IL1A*0.2414

Finally, risk factors were visually represented using the ggplot2 package in R, based on the LASSO Risk Score (Fig 7D). The findings indicated that the high-risk groups had a higher average mortality rate compared to the low-risk groups and showed elevated expression of critical genes identified by the prognostic risk model.

Utilizing the LASSO Risk Score together with the TCGA-COAD data, we conducted a Kaplan-Meier (KM) survival analysis, categorizing based on median OS rates within the COAD cohort (Fig 7E). This analysis revealed significant statistical variations in OS between the COAD groups and between the different risk categories (P < 0.001). Additionally, time-dependent ROC curves were generated for the TCGA-COAD cohort (Fig 7F), showing that the COAD prognostic risk model’s accuracy was modest over 1, 3, and 5 years (0.7 > AUC > 0.5).

### 3.8. COAD prognostic risk model prognostic analysis

**Fig 8:**
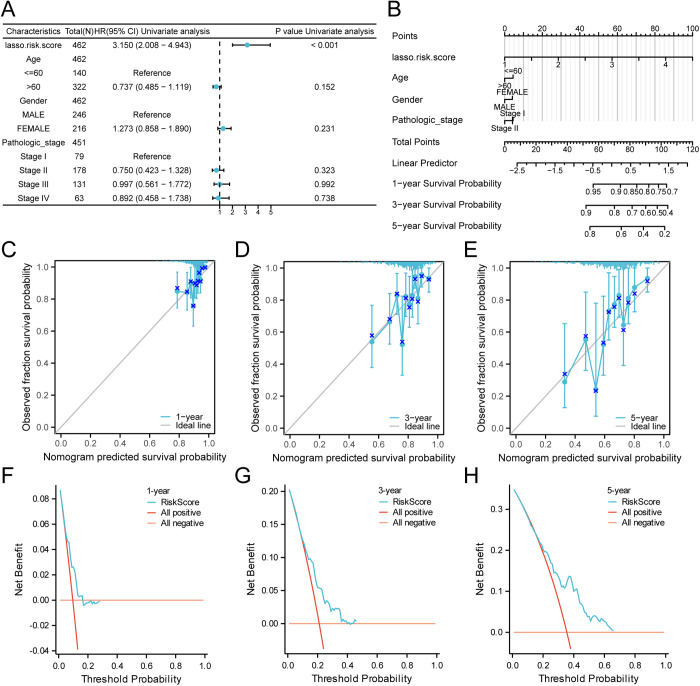
Prognostic analysis. A-b. Forest Plot (A) and Nomogram (B) of the LASSO RiskScore and clinical information in the multivariate Cox regression model. C-E. Calibration curves for 1-year (C), 3-years (D), and 5-years (E) of the prognostic risk model for COAD. F-G. 1-year (F), 3-years (G), and 5-years (H) DCA plot of the COAD prognostic risk model. TCGA, The Cancer Genome Atlas; COAD, Colon Adenocarcinoma; DCA, Decision Curve Analysis; LASSO, Least Absolute Shrinkage and Selection Operator.

Following the LASSO regression evaluation, a multivariate Cox regression analysis was applied to explore the association between risk score expression levels and the prognosis in COAD, including its predictive power. The findings were displayed using a forest plot (Fig 8A). Additionally, to further assess the efficacy of the prognostic risk model for COAD, a nomogram incorporating the LASSO Risk Score and clinical data was constructed to illustrate gene correlations (Fig 8B). The findings indicate that the LASSO Risk Score is considerably more useful than the other factors in the prognostic risk model of COAD.

Furthermore, the prognostic risk model for COAD was calibrated at three time intervals: 1-year, 3-years, and 5-years, illustrated in a calibration curve (Fig 8C–E). On this curve, the x-axis depicts the model’s expected survival probability, and the y-axis shows the actual observed survival rates. This calibration indicated that the model predictions at different time points closely matched the ideal trajectory, suggesting improved predictive accuracy, particularly at the 5-year mark. DCA was performed to demonstrate the clinical utility of the COAD predictive risk model over periods of 1-year, 3-years, and 5-years (Fig 8F–H). The model’s performance was deemed superior when its curve exceeded both the All-positive and All-negative reference lines across specified intervals. The magnitude of this improvement correlated with the range’s extent. Analytical results confirmed that our multivariate Cox regression model effectively predicted clinical outcomes, showing progressively greater accuracy at longer intervals.

### 3.9. Validation of Key Gene differential expression and ROC curve analysis

**Fig 9:**
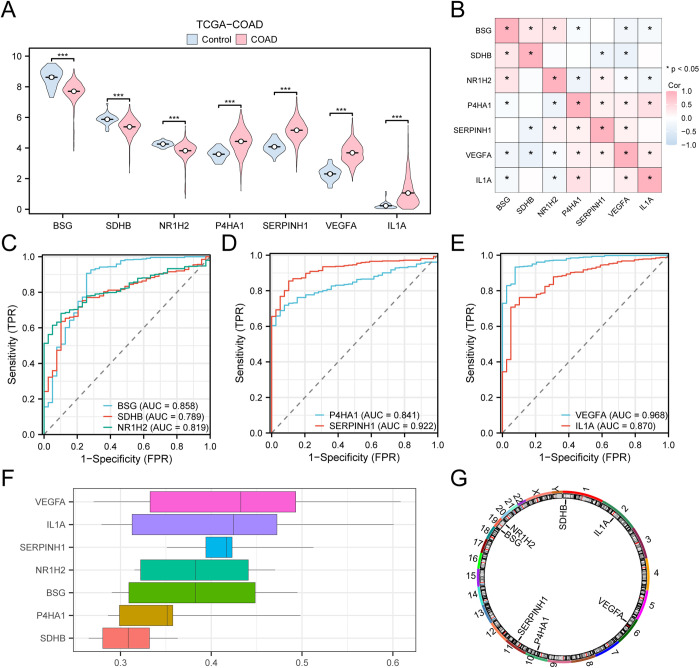
Differential expression validation and ROC curve analysis of key genes. A. Group comparison diagram of key genes in COAD specimens and control specimens in the colon adenocarcinoma dataset (TCGA-COAD). B. Correlation heatmap between key genes in the colon adenocarcinoma dataset (TCGA-COAD). ROC curves of C-E key genes *BSG, SDHB* and *NR1H2* (C), *P4HA1* and *SERPINH1* (D), *VEGFA* and *IL1A* (E) in the colon adenocarcinoma dataset (TCGA-COAD). F. Functional similarity map of key genes. G. Chromosomal mapping of key genes. *** represents *P* < 0.001, highly statistically significant. When AUC > 0.5, it indicates that the expression of the molecule is a trend to promote the occurrence of the event, and the closer the AUC is to 1, the better the diagnostic effect. AUC between 0.7 and 0.9 had a certain accuracy, and AUC above 0.9 had a high accuracy. TCGA, The Cancer Genome Atlas; COAD, Colon Adenocarcinoma; ROC, Receiver Operating Characteristic; AUC, Area Under the Curve; TPR, True Positive Rate; FPR, False Positive Rate. Group comparison plot: Light red represents COAD samples, and light blue represents Control samples. Red represents a positive correlation, and blue represents a negative correlation. The depth of the color represents the strength of the correlation. The absolute value of the correlation coefficient (r value) between 0.3 and 0.5 was a weak correlation.

The group comparison figure (Fig 9A) displays the difference analysis results of 7 key genes in the expression levels of COAD specimens and control specimens in the colon cancer dataset (TCGA-COAD), allowing researchers to investigate the key genes differential expression in the TCGA-COAD. The differential outcome displays that 7 key genes (*BSG, SDHB, NR1H2, P4HA1,* and *SERPINH1) and* the expression levels of *VEGFA* and *IL1A* in COAD specimens and control specimens in the TCGA-COAD showed statistical differences (p < 0.001).

Correlation analysis was performed on seven critical genes within the TCGA-COAD dataset, along with the creation of a correlation heatmap (Fig 9B). *IL1A* and *P4HA1* exhibited the most substantial positive correlation (r = 0.39, p < 0.05), while the most pronounced negative correlation was observed between *SDHB* and *VEGFA* (r = −0.34, p < 0.05).

Finally, ROC curves were generated using the R package pROC based on the expression levels of essential genes from the TCGA-COAD (Fig 9C–E). The two key genes in the expression levels, *SERPINH1* and *VEGFA*, were found to have high accuracy (AUC > 0.9) in the classification of COAD specimens and control specimens, according to the ROC curve. In order to differentiate among COAD specimens and control specimens, the five key genes in the expression levels (*BSG, SDHB, NR1H2, P4HA1*, and *IL1A*) showed clear accuracy (0.7 < AUC < 0.9).

The functional similarity scores obtained from the Friends analysis were utilized to identify the genes that have significant involvement in the BP of COAD (Fig 9F). According to the data, VEGFA was the gene that was closest to the critical value (cut-off = 0.60) and had a significant role in COAD.

The RCircos R program was utilized to evaluate the genomic position of 7 key genes on the human chromosome and generate a map illustrating their chromosomal localization (Fig 9G). The chromosome mapping showed that more key genes were located on chromosome 19, namely *BSG* and *NR1H2, which* were located on chromosome 19.

**Fig 10:**
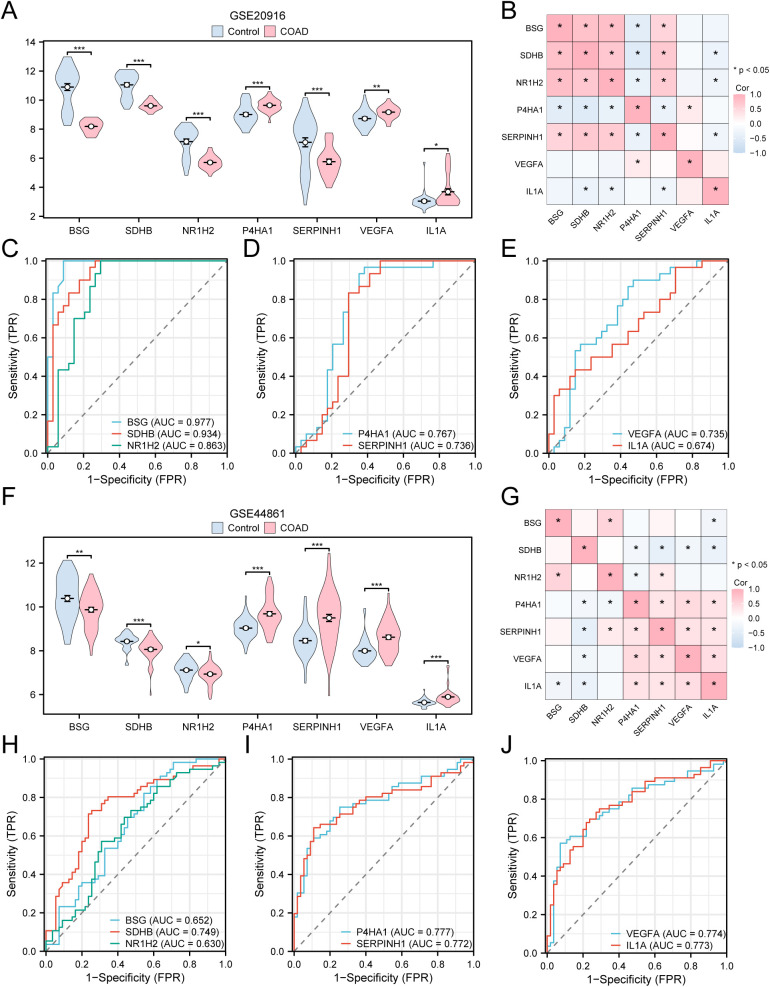
Differential expression validation and ROC curve analysis of key genes. A. Group comparison diagram of key genes in COAD specimens and control specimens of dataset GSE20916. B. Correlation heatmap between key genes in dataset GSE20916. C-E. ROC curves of key genes *BSG, SDHB* and *NR1H2* (C), *P4HA1* and *SERPINH1* (D), *VEGFA* and *IL1A* (E) in dataset GSE20916. F. Group comparison diagram of key genes in COAD specimens and control specimens in dataset GSE44861. G. Correlation heatmap between key genes in dataset GSE44861. H-j. ROC curves of key genes *BSG, SDHB* and *NR1H2* (H), *P4HA1* and *SERPINH1* (I), *VEGFA* and *IL1A* (J) in dataset GSE44861. * stands for *P* < 0.05, indicating statistical significance; ** represents *P* < 0.01, highly statistically significant; *** represents *P* < 0.001, highly statistically significant. When AUC > 0.5, it indicates that the expression of the molecule is a trend to promote the occurrence of the event, and the closer the AUC is to 1, the better the diagnostic effect. AUC between 0.5 and 0.7 had low accuracy, AUC between 0.7 and 0.9 had moderate accuracy, and AUC above 0.9 had high accuracy. TCGA, The Cancer Genome Atlas; COAD, Colon Adenocarcinoma; ROC, Receiver Operating Characteristic; AUC, Area Under the Curve; TPR, True Positive Rate; FPR, False Positive Rate. Group comparison plot: light red represents COAD samples, and light blue represents Control samples. Red represents a positive correlation, and blue represents a negative correlation. The depth of the color represents the strength of the correlation. The absolute value of the correlation coefficient (r value) between 0.3 and 0.5 was weak correlation, and between 0.5 and 0.8 was moderate correlation.

The group comparison figure (Fig 10A) displays the differential analysis results of the 7 key genes in the expression levels in COAD specimens and control specimens in dataset GSE20916, allowing for the exploration of expression differences. The differential analysis identified that the levels of expression of 5 crucial genes (*BSG*, *SDHB*, *NR1H2*, *P4HA1*, and *SERPINH1*) in COAD specimens and control specimens from dataset GSE20916 were highly statistically significant (p < 0.001).

Subsequently, we conducted correlation analysis and generated a correlation heat map to look into the expression of 7 key genes in dataset GSE20916 (Fig 10B). Among these genes, *NR1H2* and *BSG* exhibited the highest positive association (r = 0.79, *P* < 0.05). Notably, *SDHB* and *P4HA1* demonstrated a significant negative correlation, with r = −0.53 and p < 0.05.

Lastly, the expression levels of significant genes in dataset GSE20916 were analyzed with ROC curves generated by the R package pROC (Fig 10C–E). These curves revealed that *BSG* and *SDHB* had high classification accuracy (AUC > 0.9) between COAD and control specimens. The four key genes (*NR1H2*, *P4HA1*, *SERPINH1,* and *VEGFA*) expression levels showed a certain accuracy in the classification of COAD specimens and control specimens (0.7 < AUC < 0.9). The expression level of the gene *IL1A* exhibited moderate accuracy (0.5 < AUC < 0.7) when used to classify samples of COAD and Control.

To look into the variation in expression of specific genes, known as key genes, in the dataset GSE44861, a group comparison figure (Fig 10F) displays the outcomes of the study of 7 key genes in the expression levels in COAD specimens and control specimens within the GSE44861 dataset. The outcomes of the differential analysis showed that the expression levels of five key genes (*SDHB*, *P4HA1*, *SERPINH1*, *VEGFA*, and *IL1A*) in COAD samples from the GSE44861 dataset were highly statistically noteworthy in contrast to the control samples (*P* < 0.001).

Correlation assessments and heatmap visualization were conducted for seven pivotal genes within the GSE44861 dataset (Fig 10G). The strongest positive correlation occurred between *NR1H2* and *BSG* (r = 0.52, p < 0.05), whereas the most significant negative correlation was between *SDHB* and *SERPINH1* (r = −0.39, p < 0.05).

Moreover, the R package pROC was used to plot ROC curves for key gene expressions in the GSE44861 dataset (Fig 10H–J). These curves indicated moderate classification accuracy for five key genes (*SDHB, P4HA1, SERPINH1, VEGFA, IL1A*) with AUC values between 0.7 and 0.9. In contrast, the genes *BSG* and *NR1H2* displayed lower accuracy, with AUC values ranging from 0.5 to 0.7.

### 3.10. GSVA

**Fig 11:**
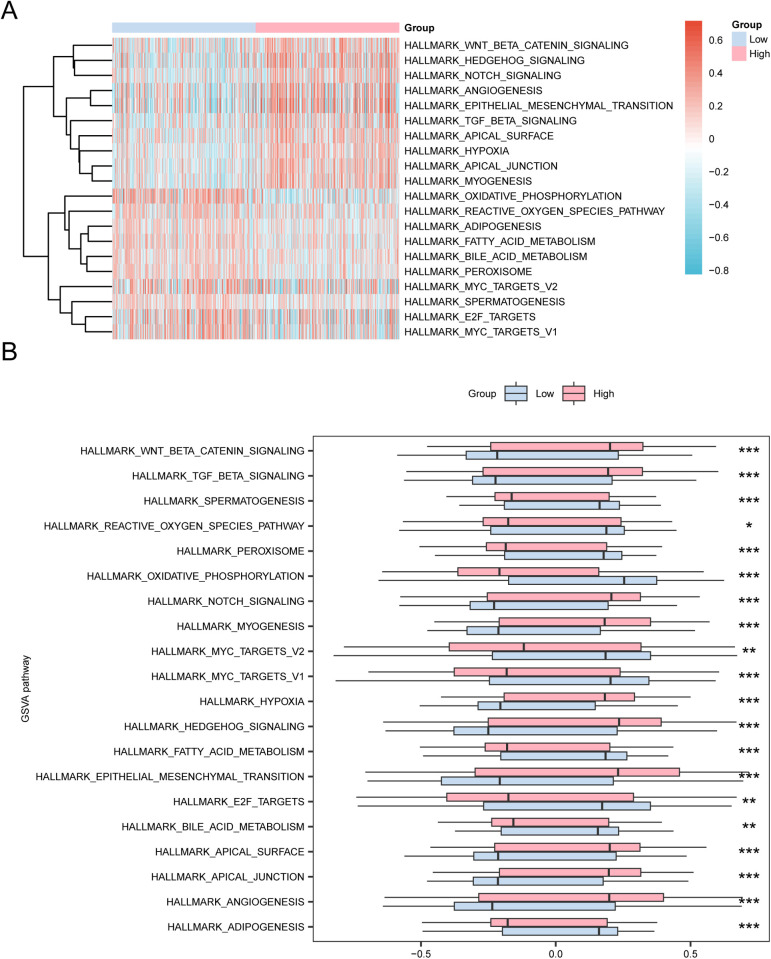
GSVA. A-B. Heat map (A) and group comparison map (B) of gene set variation analysis (GSVA) results between low- and high-risk groups of the colon adenocarcinoma dataset (TCGA-COAD). TCGA, The Cancer Genome Atlas; COAD, Colon Adenocarcinoma; GSVA, Gene Set Variation Analysis. * represents *P* < 0.05, statistically significant; ** represents *P* < 0.01, highly statistically significant; *** represents *P* < 0.001, highly statistically significant. The light red represents the high-risk groups, and the light blue represents the low-risk groups. Blue represents low enrichment, and red represents high enrichment in the heat map. The screening criterion for gene set variation analysis (GSVA) was *P* < 0.05.

To investigate differences in the h.all.v7.4.symbols.gmt gene set between low- and high-risk groups in the colon carcinoma dataset (TCGA-COAD), GSVA was performed on all the genes. The results are compiled in Table 5. Pathways with positive enrichment and significant differences (p < 0.05), ranking in the top 10 based on logFC, were highlighted. Likewise, the top 10 negatively enriched pathways were identified. The analysis of differential expression across these 20 pathways was visualized in a heat map (Fig 11A).

**Table 5:** Results of GSVA for TCGA-COAD.

ID	logFC	AveExpr	t	p value	adj.P.Val	B
HALLMARK_HEDGEHOG_SIGNALING	0.216925	0.01158	6.956896	1.15 e-11	1.15 e-10	15.75359
HALLMARK_EPITHELIAL_MESENCHYMAL_TRANSITION	0.212348	0.00782	5.814151	1.12 e-08	6.98 e-08	9.059244
HALLMARK_MYOGENESIS	0.211233	0.00853	8.046906	6.79 e-15	1.13 e-13	23.03678
HALLMARK_APICAL_JUNCTION	0.195991	0.00679	7.725636	6.61 e-14	8.27 e-13	20.80405
HALLMARK_HYPOXIA	0.195785	0.01505	8.330805	8.59 e-16	2.15 e-14	25.06711
HALLMARK_NOTCH_SIGNALING	0.175233	0.03016	6.643703	8.36 e-11	6.97 e-10	13.8192
HALLMARK_ANGIOGENESIS	0.1718	0.01945	5.091892	5.11 e-07	1.83 e-06	5.365709
HALLMARK_WNT_BETA_CATENIN_SIGNALING	0.162383	0.00318	5.773804	1.40 e-08	7.14 e-08	8.841619
HALLMARK_APICAL_SURFACE	0.155665	0.0045	5.882764	7.60 e-09	5.43 e-08	9.43235
HALLMARK_TGF_BETA_SIGNALING	0.147568	0.02772	5.154669	3.73 e-07	1.43 e-06	5.669502
HALLMARK_BILE_ACID_METABOLISM	0.06209	0.00083	2.80814	0.005187	0.009606	3.31795
HALLMARK_REACTIVE_OXYGEN_SPECIES_PATHWAY	0.06389	0.00642	2.48246	0.01339	0.023912	4.16419
HALLMARK_ADIPOGENESIS	0.08311	0.0157	3.78298	0.000175	0.000416	0.18872
HALLMARK_SPERMATOGENESIS	0.09161	0.0004	4.36088	1.59 e-05	4.67 e-05	2.077378
HALLMARK_FATTY_ACID_METABOLISM	0.10175	0.000728	4.21774	2.95 e-05	8.20 e-05	1.488218
HALLMARK_MYC_TARGETS_V2	0.1031	0.009693	2.96822	0.003146	0.00605	2.86507
HALLMARK_E2F_TARGETS	0.10977	5.18 e-06	3.16303	0.001661	0.00346	2.28134
HALLMARK_PEROXISOME	0.12055	0.00847	5.4218	9.39 e-08	3.91 e-07	6.999238
HALLMARK_MYC_TARGETS_V1	0.14518	0.00021	4.59643	5.51 e-06	1.72 e-05	3.086272
HALLMARK_OXIDATIVE_PHOSPHORYLATION	0.26819	0.00216	9.27842	6.00 e-19	3.00 e-17	32.21326

GSVA, Gene Set Variation Analysis; TCGA, The Cancer Genome Atlas; COAD, Colon Adenocarcinoma.

The differences were confirmed using the Mann-Whitney U test, followed by the creation of a comparison diagram to illustrate these disparities (Fig 11B). GSVA analysis highlighted significant differences in several pathways, including Hallmark Hedgehog Signaling, Hallmark Epithelial Mesenchymal Transition, Hallmark Myogenesis, Hallmark Apical Junction, Hallmark Hypoxia, Hallmark Notch Signaling, Hallmark Angiogenesis, Hallmark Wnt Beta Catenin Signaling, Hallmark Apical Surface, Hallmark TGF Beta Signaling, Hallmark Bile Acid Metabolism, Hallmark Reactive Oxygen Species Pathway, Hallmark Spermatogenesis, Hallmark Fatty Acid Metabolism, The Hallmark Myc Targets V2, Hallmark E2F Targets, Hallmark Peroxisome, Hallmark Myc Targets V1, and Hallmark Oxidative Phosphorylation gene sets among the low- and high-risk groups (P < 0.05).

### 3.11 Immune infiltration analysis of ssGSEA algorithm in high and low groups

**Fig 12:**
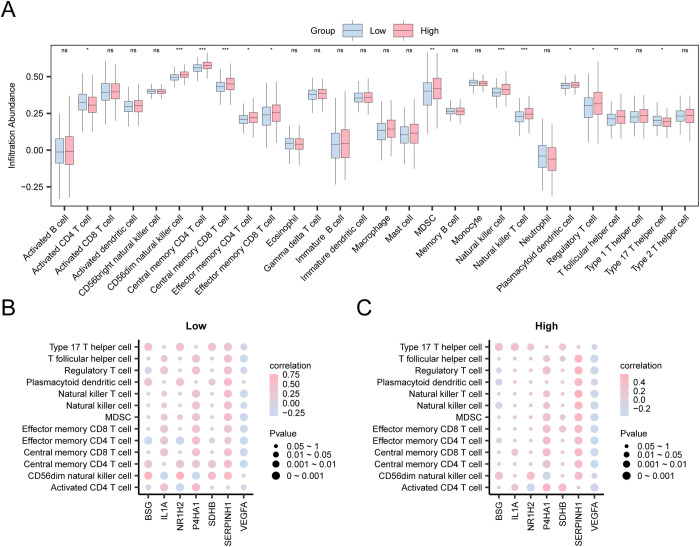
Risk Groups Immune Infiltration Analysis by ssGSEA Algorithm. A. Comparison of groupings of immune cells in the COAD group in low- and high-risk groups in the colon adenocarcinoma dataset (TCGA-COAD). B-C. Correlation bubble plots of immune cell infiltration abundance and key genes in the Low Risk (B) and High Risk (C) groups of COAD. TCGA, The Cancer Genome Atlas; COAD, Colon Adenocarcinoma; ssGSEA, Single-Sample Gene-Set Enrichment Analysis. ns stands for *P* ≥ 0.05, not statistically significant; * represents *P* < 0.05, statistically significant; ** represents *P* < 0.01, highly statistically significant; and *** represents *P* < 0.001, highly statistically significant. The absolute value of the correlation coefficient (r value) ranged from 0.5 to 0.8, indicating a moderate correlation. The light blue is for the low-risk groups, and the light red is for the high-risk groups in the group comparison. In the correlation bubble plot, light red indicates a positive correlation, and light blue indicates a negative correlation. The depth of the color represents the strength of the correlation.

Utilizing the ssGSEA method, the expression matrix of COAD specimens from TCGA-COAD was analyzed to evaluate the profiles of 28 immune cells within low- and high-risk COAD groups. A comparative plot initially showcased the variance in immunocyte infiltration levels across these groups. The analysis, illustrated in the comparison chart (Fig 12A), revealed that 13 immune cell types, including activated CD4 + T cells, central memory CD4 and CD8 T cells, effector memory CD4 and CD8 T cells, regulatory T cells, T follicular helper cells, type 17 T helper cells, CD56dim NK cells, MDSCs, NK cells, NKT cells, and plasmacytoid dendritic cells, were significantly different (P < 0.05). Lastly, the correlation between immunocyte infiltration and key genetic markers was visualized using bubble plots (Fig 12B–C). These plots revealed strong positive correlations between key genes and immunocytes within both risk groups for COAD. Specifically, *NR1H2* was strongly correlated with CD56 dim NK cells in the low-risk group (r = 0.761, P < 0.05), while *SERPINH1* displayed the most pronounced correlation with NKT cells in the high-risk group (r = 0.584, P < 0.05).

### 3.12. IPS analysis

Initially, COAD samples from the TCGA database were sorted into two categories based on the median LASSO RiskScore. Samples with a RiskScore above the median were classified as high-risk, while those with a score below the median were designated as low-risk.

**Fig 13:**
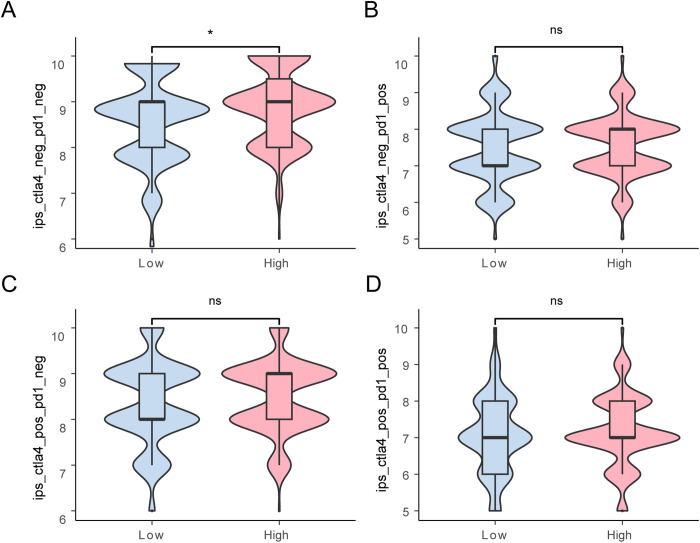
IPS analysis. A-D. Group comparison of immunogenicity score (IPS) in the low- and high-risk groups of COAD samples from the Colon Adenocarcinoma Dataset (TCGA-COAD). TCGA, The Cancer Genome Atlas; COAD, Colon Adenocarcinoma; IPS, Immunophenoscore. ns stands for *P* ≥ 0.05, not statistically significant; * represents *P* < 0.05 and statistically significant. Light red represents the high-risk groups, and light blue represents the low-risk groups.

To validate the efficacy of immunotherapy across these risk-defined groups, we retrieved the Immunophenoscore (IPS) for COAD samples from the TCIA database. Using ggplot2 in R, we generated visualizations comparing the IPS across the high- and low-risk categories defined by the LASSO RiskScore (Fig 13A–D). Significant variations in IPS were observed between these groups, indicating distinct immunological profiles (p < 0.05).

## 4. Discussion

Because of the complex pathophysiology of COAD, which is impacted by both environmental and genetic factors, it is imperative that advanced research be done to understand its molecular underpinnings. This effort could potentially facilitate early detection, targeted therapy, and enhanced patient outcomes [31].

Through an examination of gene enrichment patterns in COAD, it was determined that DEGs related to glycolysis and pyroptosis play a key role in tumor growth and response to treatment. Glycolysis, often upregulated in cancer cells, is a hallmark of the metabolic reprogramming that supports rapid cell proliferation and survival under hypoxic conditions, a phenomenon known as the Warburg effect [32]. GRGs and PRGs have been implicated in various cancers, influencing tumor growth, metastasis, and resistance to therapy [33]. For instance, studies have shown that targeting glycolysis can significantly impair cancer cell proliferation, suggesting that GRGs may serve as potential therapeutic targets [34]. Our study identified 53 DEGs (G&PRDEGs) associated with glycolysis and pyroptosis in the TCGA-COAD, encompassing genes such as *BAK1, BCL2*, and *BRCA1*. The LASSO regression model identified seven key genes (key genes): *BSG, SDHB, NR1H2, P4HA1, SERPINH1, VEGFA*, and *IL1A*. The univariate Cox regression analysis revealed a substantial association between these critical genes and the prognosis of COAD sufferers. These genes were considered crucial variables for developing the prognostic risk model in the LASSO regression study. These genes exhibit significant differences between the COAD group and the control group, closely linking them to the glycolysis and pyroptosis processes—two crucial biological pathways in tumor development and treatment. In COAD, high expression of SDHB enhances patient prognosis [35]. Furthermore, P4HA1 is highly expressed in CRC tissues and cells, and knocking down P4HA1 inhibits CRC cell proliferation, induces cell cycle arrest at the G0/G1 phase, decreases CRC cell stemness, reduces tumor sphere formation and size, lowers CRC cell chemoresistance, and increases Caspase-3 activity [36]. Research shows that SERPINH1 promotes CRC proliferation and metastasis by activating the PI3K/Akt/mTOR pathway [37]. This study established a COAD risk score model based on composite expression levels of seven key genes and confirmed that this model demonstrated good discriminative power in patient prognosis stratification. Further analysis revealed that although patients with the poorest prognosis showed the highest co-expression of all seven key genes, most patients in clinical practice exhibit partial gene expression positivity. Group survival analysis showed that as the number of high-risk gene expressions increased, overall patient survival rates progressively declined, and certain survival differences between different gene combinations. This finding suggests that multi-gene co-expression and partial expression have important clinical implications for predicting risk stratification of COAD patients. In clinical practice, conducting more detailed personalized risk assessments based on different gene expression combinations can provide guidance to formulating more precise treatment strategies. Future related research could further integrate large-scale multicenter clinical data to explore the mechanisms of how different gene combinations affect the prognosis of COAD and their clinical utility in precision medicine.

The GO and KEGG pathway enriching studies emphasized the relationship between metabolic reprogramming and cell death pathways in COAD. For example, pathways like the ‘HIF-1 signaling pathway’ were enriched for their involvement in hypoxia response and cell cycle regulation, respectively [38]. These pathways are critical for understanding the adaptation of cancer cells to their environment and the potential vulnerabilities that could be targeted therapeutically. The GO and KEGG analyses in our study revealed that 53 G&PRDEGs associated with glycolysis and pyroptosis were predominantly enriched in various BP, including oxidative stress response and regeneration system development, along with multiple signaling pathways such as lipid metabolism and atherosclerosis. These analyses unveiled crucial BP and pathways in which G&PRDEGs may be involved in COAD. For instance, the oxidative stress response has been linked to the advancement of certain forms of cancer and can be utilized as a therapeutic goal.

The prognostic significance of immune cell infiltration has been increasingly recognized, with studies [39] showing that the presence and type of immune cells in the TME can act as biomarkers to anticipate patient outcomes and responses to treatments. For instance, a high density of tumor-infiltrating immunological cells has been connected to a favorable prognosis in various cancers, including COAD [40]. In contrast, our research indicates that the high-risk groups, with a higher expression of key genes and a greater number of adverse events, exhibit a distinct immune cell infiltration profile, which could reflect a more immunosuppressive or immune-evasive TME.

The article emphasizes the essential significance of the immune landscape in predicting the prognosis of COAD sufferers by demonstrating significant differences in immunocyte infiltration among low- and high-risk groups. Immunocytes located inside the microenvironment of the tumor [41], such as B cells, T cells, macrophages, and dendritic cells, are known to affect cancer cell progression and patient survival. For instance, cytotoxic T cells [42] can exert anti-tumor effects by recognizing and destroying cancer cells, while regulatory T cells can restrain the immune effects, potentially aiding tumor escape. Our findings align with the notion that a higher immune infiltration is often associated with a better prognosis in COAD [43], as it may reflect a more active anti-tumor immune response. The study’s investigation of the amount of immunocyte infiltration revealed substantial differences among 13 categories of immunocytes between the high- and low-risk groups. The presence of immunocytes in a tumor’s microenvironment is a crucial component that impacts both the prognosis of patients and the overall condition of the body’s immune response to the tumor. The levels of immunocyte infiltration can indicate the status of the DY immunity response to the tumor and potentially influence the patient’s response to immunotherapy. In conclusion, our immune analysis results aid in raising understanding of the reciprocity among the immune system and COAD, highlighting the potential of immune-related biomarkers in predicting patient prognosis and tailoring immunotherapeutic interventions. Integrating immune profiling with risk stratification models may facilitate the development of personalized and efficacious cancer treatments [44].

Although our study is extensive, it is crucial to recognize specific constraints that could affect the understanding of our results. Firstly, our research was primarily computational and did not include wet-lab experiments to validate the bioinformatics predictions. This could potentially limit the translational applicability of our results. Secondly, the sample size, although adequate for initial discovery, might not be sufficient to capture the full spectrum of genetic variability present in COAD. Thirdly, the lack of extensive clinical validation analysis means that the prognostic models and biomarkers identified need further confirmation in prospective studies. Finally, the utilization of numerous datasets brings about the potential for batch effects, even though we have made attempts to minimize these through normalization and batch correction processes. These drawbacks emphasize the necessity of more study to validate and broaden our conclusions.

## 5. Conclusion

Our study effectively identified genes that are differently expressed and associated with glycolysis and pyroptosis in COAD. We also investigated SM and CNV. Additionally, we gave insights into the biological processes and pathways. The evolution of a predictive risk model and the assessment of immune infiltration and immunogenicity scores signify substantial advancements in comprehending the intricate interaction between COAD genetics and the tumor microenvironment. The results of this research could possibly help in the creation of novel therapeutic modalities and enhance the accuracy of predicting outcomes. In the future, translating these computational findings into real-world benefits for COAD patients will require integrating them with experimental validation and clinical trials. However, the exact pathophysiology and molecular targets require further confirmation.

## Supporting information

S1 Table Glycolysis & Pyroptosis-Related Genes list. (PDF)

S2 Table G&PRDEGs. (PDF)

S1 File Additional data1. (ZIP)

S2 File Additional data2. (ZIP)

S3 File Additional data3. (ZIP)
