# Supplementary material for: Mechanistic investigation of glycolysis and pyroptosis in colon adenocarcinoma tissues, and prognostic analysis of patient clinical outcomes
Source: PLoS One. 2025 Jul 18;20(7):e0328560. doi: 10.1371/journal.pone.0328560 (PMC12273967; doi:10.1371/journal.pone.0328560)
Supplement: S1 Table — (PDF) [file pone.0328560.s001.pdf]

S1 Table. Glycolysis & Pyroptosis-Related Genes list

TP53
MTOR
SIRT6
AKT1
PPARG
BSG
SDHB
STAT3
HKDC1
TUG1
SLC16A4
NFE2L2
SIRT3
PARP1
HSP90B1
HNRNPA2B1
HSF1
YWHAZ
PTEN
FASN
FOXO3
EZH2
SIRT1
RELA
TRAF6
HSP90AA1
TRIM21
METTL3
TXNIP
NFKB1
IL6
BRCA1
PRMT5
GSTP1
MUC1
HMGB1
TNF
VIM
HSP90AB1
PRKN
TP63
CD274
IL1B
KLF4
RPL7A
ANXA2
HDAC2
ETS1

NR4A1
RIPK1
PRDX6
BCL2
APP
SQSTM1
ALK
ULK1
JUN
MRE11
DDX3X
H2AC14
CUL4B
NLRP3
LMNA
NOS2
MYH9
YWHAE
MDM2
BAX
FOXP3
APOE
BRD4
TFAP2A
CXCL8
ADORA1
BECN1
H2AX
RRBP1
FUS
FTO
H4C1
H2BC3
TRIM31
AGER
TUBB6
RBBP7
TSLP
CEBPB
ELAVL1
IL27
NDUFA13
H3-3B
H2BC1
H1-5
H4C9
ACTN4
MAPK14
CASP1
BIRC2

P2RX7
DNMT3B
CSNK1A1
ROCK1
STAT2
CDK9
SRPK1
IGF2BP3
BHLHE40
DEPTOR
CYCS
MAPT
IFIH1
CALM1
SPTBN1
IQGAP1
RPL3
H2BC21
H2AZ2
H4C3
ATOH8
STXBP1
UCP1
GSK3B
BNIP3
ADIPOQ
LRPPRC
DHX9
S100A8
PINK1
S100A9
RBMX
ACTG1
H2BC8
H1-2
H2AC20
TFAM
SYVN1
CASP3
TLR4
DNMT3A
TLR3
CDKN1B
USP9X
GATA6
HUWE1
NEDD4
STAT5A
TOMM20
GPX4

PRG2
RUNX2
HP
FNDC5
GJA1
CASP8
NLRX1
CYBB
ANXA1
ADORA2A
NR1H2
USP24
BNIP3L
H3C1
MMP9
ABL1
IRF1
SPP1
TNFSF13B
ATG7
CCL5
HNRNPC
FGF21
H2BC11
H4C14
PTGS2
IKBKG
NOS1
USP8
TET2
S100A4
MKI67
H3-3A
GPER1
SETD7
METTL14
TRPM2
H3C12
H3C13
H3C14
H3C2
H3C4
H3C11
H3C3
H3C6
H3C10
H3C8
H3C7
H3C15
DNMT1

TLR2
CASP9
CFH
VCAM1
FADD
BAK1
PLAUR
CHMP2B
AIM2
USP25
H4C11
H4C6
H2BC14
H4C16
H4C5
H2BC13
H2BC9
H4C8
H2BC5
H4C2
H4C4
H2BC12
H2BC15
H2BC4
H2BC6
H4C12
H2BC10
H2BC17
H2BC26
H4C13
H2BC7
H4C15
ACE2
LDLR
APAF1
CHI3L1
IL17A
PKN2
PECAM1
PAK2
EEF2K
FMR1
TRAF2
CRTAC1
NFKBIA
ATG5
IL1A
TRIM24
RIPK3
TLR8

SERPINH1
RAB5A
RBBP4
FSTL1
USP48
GSDMD
H2AC4
EPHA2
AXL
APOA1
ATF6
CASP7
DRD2
PLCG1
SPTAN1
VDR
TPM3
TRAF3
FPR2
IRF3
LCN2
PANX1
TREM2
ASIC1
EED
IL13
VTN
PTX3
SUZ12
USP14
STING1
PDCD6IP
PVALB
TNFRSF21
USF2
AHSA1
GNA15
IFI27
IL32
RPL27A
SSR1
GSDME
ORMDL3
YTHDF2
RSL1D1
SEC22B
GSDMA
GSDMC
USP47
CITED2

CASP6
PKM
ADORA2B
EGFR
MDH1
P4HA1
VEGFA
CDK1
GAPDH
PRKACA
