# Supplementary material for: Mechanistic investigation of glycolysis and pyroptosis in colon adenocarcinoma tissues, and prognostic analysis of patient clinical outcomes
Source: PLoS One. 2025 Jul 18;20(7):e0328560. doi: 10.1371/journal.pone.0328560 (PMC12273967; doi:10.1371/journal.pone.0328560)
Supplement: S2 Table — (PDF) [file pone.0328560.s002.pdf]

S2 Table. G&PRDEGs

STXBP1
TFAP2A
CITED2
BSG
LCN2
NDUFA13
BAK1
DNMT3B
SLC16A4
SDHB
TXNIP
NEDD4
CCL5
PINK1
HDAC2
NFKBIA
FASN
HSP90B1
GSTP1
CYCS
NR1H2
TRIM24
KLF4
VDR
PRDX6
HSP90AB1
SRPK1
CFH
TLR8
MMP9
P4HA1
BRCA1
GNA15
DNMT1
TOMM20
RBBP7
TRPM2
SERPINH1
CEBPB
VEGFA
TLR3
CHI3L1
MKI67
IL1A
IGF2BP3
PPARG
HMGB1
PECAM1

HSP90AA1
CASP7
IL6
IL1B
BCL2
