# Supplementary material for: Mechanistic investigation of glycolysis and pyroptosis in colon adenocarcinoma tissues, and prognostic analysis of patient clinical outcomes
Source: PLoS One. 2025 Jul 18;20(7):e0328560. doi: 10.1371/journal.pone.0328560 (PMC12273967; doi:10.1371/journal.pone.0328560)
Supplement: S1 File — (ZIP) [file pone.0328560.s003.zip › Additional data1/Table1.docx]

### Table 1 Overall Baseline Data Sheet

| Characteristics | Overall |
| --- | --- |
| Age，n (%) |  |
| >60 | 322（69.7%） |
| <=60 | 140（30.3%） |
| Gender，n (%) |  |
| MALE | 246（53.2%） |
| FEMALE | 216（46.8%） |
| Pathologic_stage，n (%) |  |
| Stage IV | 63（14.0%） |
| Stage II | 178（39.5%） |
| Stage I | 79（17.5%） |
| Stage III | 131（29.0%） |
