# Supplementary material for: Mechanistic investigation of glycolysis and pyroptosis in colon adenocarcinoma tissues, and prognostic analysis of patient clinical outcomes
Source: PLoS One. 2025 Jul 18;20(7):e0328560. doi: 10.1371/journal.pone.0328560 (PMC12273967; doi:10.1371/journal.pone.0328560)
Supplement: S1 File — (ZIP) [file pone.0328560.s003.zip › Additional data1/Table2.docx]

### Table 2 GEO Microarray Chip Information

|  | GSE20916 | GSE44861 |
| --- | --- | --- |
| Platform | GPL570 | GPL3921 |
| Species | Homo sapiens | Homo sapiens |
| Tissue | Colon Tissues | Colon Tissues |
| Samples in COAD group | 30 | 56 |
| Samples in Control group | 34 | 55 |
| Reference | PMID：20957034 | PMID：23982929 |

GEO，Gene Expression Omnibus；COAD，Colon Cancer。
