# Supplementary material for: Mechanistic investigation of glycolysis and pyroptosis in colon adenocarcinoma tissues, and prognostic analysis of patient clinical outcomes
Source: PLoS One. 2025 Jul 18;20(7):e0328560. doi: 10.1371/journal.pone.0328560 (PMC12273967; doi:10.1371/journal.pone.0328560)
Supplement: S1 File — (ZIP) [file pone.0328560.s003.zip › Additional data1/Table3.docx]

### Table 3 Results of GO and KEGG Enrichment Analysis for G&PRDEGs

| ONTOLOGY | ID | Description | GeneRatio | BgRatio | pvalue | p.adjust | qvalue |
| --- | --- | --- | --- | --- | --- | --- | --- |
| BP | GO:0006979 | response to oxidative stress | 11/53 | 433/18800 | 2.721E-08 | 9.6509E-06 | 4.9526E-06 |
| BP | GO:0048608 | reproductive structure development | 11/53 | 433/18800 | 2.721E-08 | 9.6509E-06 | 4.9526E-06 |
| BP | GO:1903131 | mononuclear cell differentiation | 11/53 | 433/18800 | 2.721E-08 | 9.6509E-06 | 4.9526E-06 |
| BP | GO:0061458 | reproductive system development | 11/53 | 436/18800 | 2.9201E-08 | 9.6509E-06 | 4.9526E-06 |
| BP | GO:0001819 | positive regulation of cytokine production | 11/53 | 475/18800 | 6.9819E-08 | 1.6782E-05 | 8.6122E-06 |
| CC | GO:0034774 | secretory granule lumen | 8/53 | 322/19594 | 2.2719E-06 | 0.00014434 | 9.1159E-05 |
| CC | GO:0060205 | cytoplasmic vesicle lumen | 8/53 | 325/19594 | 2.4338E-06 | 0.00014434 | 9.1159E-05 |
| CC | GO:0031983 | vesicle lumen | 8/53 | 327/19594 | 2.5471E-06 | 0.00014434 | 9.1159E-05 |
| CC | GO:0005667 | transcription regulator complex | 8/53 | 483/19594 | 4.3044E-05 | 0.00073174 | 0.00046215 |
| CC | GO:0101002 | ficolin-1-rich granule | 6/53 | 185/19594 | 1.0374E-05 | 0.00031932 | 0.00020167 |
| MF | GO:0140297 | DNA-binding transcription factor binding | 11/53 | 470/18410 | 7.7404E-08 | 1.1224E-05 | 8.0255E-06 |
| MF | GO:0061629 | RNA polymerase II-specific DNA-binding transcription factor binding | 10/53 | 348/18410 | 4.8507E-08 | 1.1224E-05 | 8.0255E-06 |
| MF | GO:0044389 | ubiquitin-like protein ligase binding | 9/53 | 317/18410 | 2.706E-07 | 2.6158E-05 | 1.8705E-05 |
| MF | GO:0031625 | ubiquitin protein ligase binding | 8/53 | 298/18410 | 2.0208E-06 | 0.00014651 | 0.00010476 |
| MF | GO:0005125 | cytokine activity | 6/53 | 235/18410 | 5.6427E-05 | 0.00163639 | 0.00117012 |
| KEGG | hsa05417 | Lipid and atherosclerosis | 12/43 | 215/8164 | 6.124E-10 | 8.0267E-08 | 4.717E-08 |
| KEGG | hsa05418 | Fluid shear stress and atherosclerosis | 10/43 | 139/8164 | 1.7568E-09 | 9.5452E-08 | 5.6094E-08 |
| KEGG | hsa05132 | Salmonella infection | 10/43 | 249/8164 | 4.5949E-07 | 1.498E-05 | 8.803E-06 |
| KEGG | hsa05022 | Pathways of neurodegeneration - multiple diseases | 10/43 | 476/8164 | 0.00013836 | 0.00112764 | 0.00066268 |
| KEGG | hsa04657 | IL-17 signaling pathway | 9/43 | 94/8164 | 9.8487E-10 | 8.0267E-08 | 4.717E-08 |

GO，Gene Ontology；BP，Biological Process；CC，Cellular Component；MF，Molecular Function；KEGG，Kyoto Encyclopedia of Genes and Genomes；G&PRDEGs，Glycolysis & Pyroptosis-Related Differentially Expressed Genes。
