# Supplementary material for: Mechanistic investigation of glycolysis and pyroptosis in colon adenocarcinoma tissues, and prognostic analysis of patient clinical outcomes
Source: PLoS One. 2025 Jul 18;20(7):e0328560. doi: 10.1371/journal.pone.0328560 (PMC12273967; doi:10.1371/journal.pone.0328560)
Supplement: S1 File — (ZIP) [file pone.0328560.s003.zip › Additional data1/Table4.docx]

### Table 4 Results of GSEA for TCGA-COAD

| ID | setSize | enrichmentScore | NES | pvalue | p.adjust | qvalue |
| --- | --- | --- | --- | --- | --- | --- |
| REACTOME_INFLUENZA_INFECTION | 156 | 0.50752594 | 2.24052592 | 1E-10 | 6.6215E-09 | 4.924E-09 |
| WP_PHOTODYNAMIC_THERAPYINDUCED_NFKB_SURVIVAL_SIGNALING | 34 | 0.62274719 | 2.04903893 | 6.4721E-05 | 0.00104873 | 0.00077988 |
| REACTOME_SELENOAMINO_ACID_METABOLISM | 117 | 0.46855192 | 1.96010399 | 8.9576E-07 | 2.4789E-05 | 1.8434E-05 |
| REACTOME_ASSEMBLY_OF_COLLAGEN_FIBRILS_AND_OTHER_MULTIMERIC_STRUCTURES | 61 | 0.50260173 | 1.86783658 | 0.00031935 | 0.00390793 | 0.00290608 |

TCGA，The Cancer Genome Atlas；COAD，Colon Cancer；GSEA，Gene Set Enrichment Analysis。
