# Supplementary material for: Mechanistic investigation of glycolysis and pyroptosis in colon adenocarcinoma tissues, and prognostic analysis of patient clinical outcomes
Source: PLoS One. 2025 Jul 18;20(7):e0328560. doi: 10.1371/journal.pone.0328560 (PMC12273967; doi:10.1371/journal.pone.0328560)
Supplement: S1 File — (ZIP) [file pone.0328560.s003.zip › Additional data1/Table5.docx]

### Table 5 Results of GSVA for TCGA-COAD

| ID | logFC | AveExpr | t | P.Value | adj.P.Val | B |
| --- | --- | --- | --- | --- | --- | --- |
| HALLMARK_HEDGEHOG_SIGNALING | 0.216925 | -0.01158 | 6.956896 | 1.15E-11 | 1.15E-10 | 15.75359 |
| HALLMARK_EPITHELIAL_MESENCHYMAL_TRANSITION | 0.212348 | -0.00782 | 5.814151 | 1.12E-08 | 6.98E-08 | 9.059244 |
| HALLMARK_MYOGENESIS | 0.211233 | -0.00853 | 8.046906 | 6.79E-15 | 1.13E-13 | 23.03678 |
| HALLMARK_APICAL_JUNCTION | 0.195991 | -0.00679 | 7.725636 | 6.61E-14 | 8.27E-13 | 20.80405 |
| HALLMARK_HYPOXIA | 0.195785 | -0.01505 | 8.330805 | 8.59E-16 | 2.15E-14 | 25.06711 |
| HALLMARK_NOTCH_SIGNALING | 0.175233 | -0.03016 | 6.643703 | 8.36E-11 | 6.97E-10 | 13.8192 |
| HALLMARK_ANGIOGENESIS | 0.1718 | -0.01945 | 5.091892 | 5.11E-07 | 1.83E-06 | 5.365709 |
| HALLMARK_WNT_BETA_CATENIN_SIGNALING | 0.162383 | -0.00318 | 5.773804 | 1.40E-08 | 7.14E-08 | 8.841619 |
| HALLMARK_APICAL_SURFACE | 0.155665 | -0.0045 | 5.882764 | 7.60E-09 | 5.43E-08 | 9.43235 |
| HALLMARK_TGF_BETA_SIGNALING | 0.147568 | -0.02772 | 5.154669 | 3.73E-07 | 1.43E-06 | 5.669502 |
| HALLMARK_BILE_ACID_METABOLISM | -0.06209 | -0.00083 | -2.80814 | 0.005187 | 0.009606 | -3.31795 |
| HALLMARK_REACTIVE_OXYGEN_SPECIES_PATHWAY | -0.06389 | -0.00642 | -2.48246 | 0.01339 | 0.023912 | -4.16419 |
| HALLMARK_ADIPOGENESIS | -0.08311 | -0.0157 | -3.78298 | 0.000175 | 0.000416 | -0.18872 |
| HALLMARK_SPERMATOGENESIS | -0.09161 | -0.0004 | -4.36088 | 1.59E-05 | 4.67E-05 | 2.077378 |
| HALLMARK_FATTY_ACID_METABOLISM | -0.10175 | 0.000728 | -4.21774 | 2.95E-05 | 8.20E-05 | 1.488218 |
| HALLMARK_MYC_TARGETS_V2 | -0.1031 | 0.009693 | -2.96822 | 0.003146 | 0.00605 | -2.86507 |
| HALLMARK_E2F_TARGETS | -0.10977 | 5.18E-06 | -3.16303 | 0.001661 | 0.00346 | -2.28134 |
| HALLMARK_PEROXISOME | -0.12055 | -0.00847 | -5.4218 | 9.39E-08 | 3.91E-07 | 6.999238 |
| HALLMARK_MYC_TARGETS_V1 | -0.14518 | -0.00021 | -4.59643 | 5.51E-06 | 1.72E-05 | 3.086272 |
| HALLMARK_OXIDATIVE_PHOSPHORYLATION | -0.26819 | -0.00216 | -9.27842 | 6.00E-19 | 3.00E-17 | 32.21326 |

GSVA，Gene Set Variation Analysis；TCGA，The Cancer Genome Atlas；COAD，Colon Cancer；。
